# Transcriptional landscape of human endogenous retroviruses (HERVs) and other repetitive elements in psoriatic skin

**DOI:** 10.1038/s41598-018-22734-7

**Published:** 2018-03-12

**Authors:** Freddy Lättekivi, Sulev Kõks, Maris Keermann, Ene Reimann, Ele Prans, Kristi Abram, Helgi Silm, Gea Kõks, Külli Kingo

**Affiliations:** 1 Department of Pathophysiology, University of Tartu, Tartu, Estonia; 2 Department of Dermatology, University of Tartu, Tartu, Estonia; 3 Clinic of Dermatology, Tartu University Hospital, Tartu, Estonia; 4 Department of Reproductive Biology, Estonian University of Life Sciences, Tartu, Estonia

## Abstract

Human endogenous retrovirus (HERV) sequences make up at least 8% of the human genome. Transcripts originating from these loci as well as proteins encoded by them have been detected in various tissues. HERVs are believed to be implicated in autoimmune diseases, however the extent to which, has remained unclear. Differential expression studies have so far been limited to certain HERV subfamilies with conserved sequences. No studies have been published describing the genome-wide expression pattern of HERVs and repetitive elements in the context of psoriasis. In the present study, we analysed total RNA sequencing data from skin samples of 12 psoriasis patients and 12 healthy controls, which enabled us to describe the entire transcriptional landscape of repetitive elements. We report high levels of repetitive element expression in the skin of psoriasis patients as well as healthy controls. The majority of differentially expressed elements were downregulated in lesional and non-lesional skin, suggesting active HERV suppression in the pro-inflammatory environment of psoriatic skin. However, we also report upregulation of a small subset of HERVs previously described in the context of autoimmune diseases, such as members of the HERV-K and W families, with the potential to affect the immunopathogenesis of psoriasis.

## Introduction

Human endogenous retroviruses (HERVs), members of the long terminal repeat (LTR) retrotransposons repetitive element class, make up at least 8–10% of the human genome^1^. Having acquired numerous mutations over time, human endogenous retroviruses (HERVs) are believed to be incapable of replicating or forming infectious particles^2^. While it has been shown that LINEs and SINEs (non-LTR retrotransposons) can be the cause of several human genetic disorders^3^, the view on HERVs is not yet as clear. Regulatory loci in HERV sequences can act as enhancers, leading to altered expression of nearby genes^4^, or as extra transcription start sites, causing the formation of chimeric transcripts^5^. Normally, HERVs and other retrotransposons are repressed by DNA methylation^6^ or intrinsic antiviral factors^7^. As exogenous retroviruses are capable of inducing immune response, the expressed HERV sequences and proteins encoded by them can also be recognized by the host immune system as foreign and potentially hostile elements^8^. For example, members of the HERV-W family have been found to activate the innate immune system via CD14/TLR4 signaling^9^.

Psoriasis is an immune-mediated chronic inflammatory disease of the skin. Implication of viral elements in the immune responses of psoriasis is not a recent idea^10,11^. More recently, expression of retroviral polymerase (pol) encoding members of the HERV-W, K and E families have been reported in psoriatic skin, while rarely detected in non-lesional skin^12^. Transcripts originating from a copy of HERV-K deoxyuridine triphosphate nucleotidohydrolase (dUTPase), located within the psoriasis susceptibility 1 (PSORS1) locus, have been found in both peripheral blood mononuclear cells (PBMCs) and skin tissue^13^. It has been demonstrated that HERV-K dUTPase proteins can induce the secretion of Th1 and Th17 cytokines in both dendric cells and keratinocytes^14^. Conversely, Gupta and colleagues demonstrated the downregulation of transcripts containing HERV-K env, gag or pol sequences in the lesional skin of psoriasis patients compared to non-lesional skin and healthy skin from control group subjects^15^.

With the exception of the study by Molès and colleagues^12^, limited to pol-encoding HERVs, no studies have been published describing the genome-wide expression pattern of HERVs and repetitive elements in the context of psoriasis. In the present study, we re-analysed our previously published RNA-seq data^16,17^, utilizing the RepEnrich pipeline^18^ in order to investigate the genome-wide transcriptional landscape of HERVs and repetitive elements in the lesional and non-lesional skin of psoriasis patients.

## Results

### RNA sequencing and read alignment

RNA sequencing of the 12 lesional skin (LP), 12 non-lesional skin (NLP) and 12 healthy control (C) samples investigated in this study produced on average 27 million reads per sample (Fig. S1). With no mapping quality cut-off, on average 68.6% of the sequenced reads mapped to the hg19 reference genome. 27.5% of reads mapping to the hg19 reference aligned to annotated repetitive elements loci in the RepeatMasker^19^ hg19 Library. Out of 1116 repetitive element subfamilies queried, we detected the expression of 1033 subfamilies with a cut-off of one or more aligned reads per kilobase of transcript per million mapped reads (RPKM ≥ 1) as mean of all samples. Each of the subfamilies represent all known genomic instances of a given repetitive element classified by RepeatMasker, and are hereinafter referred to as *repetitive elements* or simply as *elements*. RPKM values and raw counts of all expressed elements can be found in Supplementary Materials.

The expression of some repetitive elements is characterized by very large RPKM values (Fig. S2A and B). The mean RPKM values of all samples per element are in correlation (Pearson’s *r* = 1; p ≤ 2.2 * 10^−16^) with the number of annotated genomic instances for that element (Fig. S3). For example, 142 003 and 48 914 separate genomic loci are annotated for AluSx and AluSP elements with RPKM values amounting to 40 768 and 14 565, respectively. The subsequently detected differentially expressed (DE) elements were not more likely to have higher expression levels than not differentially expressed elements (p-value > 0.05, one-sided Mann-Whitney U test). No differences in the relative abundance of reads originating from different repetitive element classes were observed between study groups in our dataset (Table 1). We note that the variability of expression levels is larger in the control group compared to the LP and NLP groups (Fig. S4A–C).

**Table 1:** Relative abundance of repetitive element classes.

Element Class	LP	NLP	C	RepeatMasker
SINE	31.72	30.84	31.43	11.33
Satellite	0.09	0.10	0.14	0.35
scRNA	0.03	0.03	0.04	≤0.01
LTR	15.96	16.39	16.01	7.46
srpRNA	0.03	0.04	0.03	≤0.01
DNA	7.54	7.67	7.63	3.15
snRNA	0.05	0.05	0.05	≤0.01
RNA	0.06	0.08	0.05	≤0.01
LINE	44.48	44.75	44.56	77.68
RC	0.04	0.04	0.04	≤0.01

Columns “LP”, “NLP”, and “C” represent relative abundance of repetitive element classes based on read counts in the corresponding study groups and column “RepeatMasker” describes the relative amount of basepairs annotated per element class in RepeatMasker hg19 Library. All values presented as percentages. LP – lesional skin; NLP – non-lesional skin; C – control group.

### Differentially expressed elements: LP vs C and NLP vs C

Comparisons between LP and C, NLP and C groups resulted in 414 and 91 DE elements at FDR ≤ 0.01, respectively. Compared to the control group, most of the repetitive elements were less expressed in both the LP (98%) and NLP (95%) groups (Fig. S5). The few upregulated elements can be found in Table 2. In addition to HERV sequences, small nuclear RNAs (snRNA) U17 and U14, involved in ribosomal RNA processing^20^, and satellite repeat MSR1 were also upregulated in the LP group compared to the control group. In the NLP vs C comparison, the only upregulated non-LTR sequence was satellite repeat HSAT5. Elements differentially expressed at a level of log_2_FC ≤ −0.5 or log_2_FC ≥ 0.5 (translating into foldchange difference greater than 0.71 or 1.41) are listed in Tables S1 and S2. The full listing of differentially expressed elements can be found in Tables S3 and S4. At the repeat family level, we observed 17 and 3 downregulated families at FDR ≤ 0.01 in the LP vs C and NLP vs C comparisons, respectively (Tables S5 and S6).

**Table 2:** Upregulated repetitive elements amongst the differentially expressed elements.

Class	Family	Element	LP vs C	NLP vs C	LP vs NLP
LTR	ERVK	LTR5	0.76	1.00	**−0.23 (0.127)**
LTR	ERVK	HERVK11D-int	0.18 (0.207)	**−0.20 (0.406)**	0.38
LTR	ERVK	HERVK14C-int	0.14 (0.338)	**−0.2 (0.190)**	0.40
LTR	ERV1	MER34B-int	0.13 (0.246)	**−0.17 (0.333)**	0.29
LTR	ERV1	MER51C	0.85	0.17 (0.381)	0.64
LTR	ERV1	MER65B	0.10 (0.397)	**−0.49**	0.61
LTR	ERV1	MER65D	0.28 (0.023)	0.08 (0.777)	0.19
LTR	ERV1	MER66A	0.34	0.29 (0.090)	0.05 (0.497)
LTR	ERV1	MER84	**−0.15 (0.199)**	**−0.36**	0.21
LTR	ERV1	ERV24B_Prim-int	0.25 (0.036)	0.45	**−0.20**
LTR	ERV1	LTR10B2	**−0.59**	0.40	**−0.97**
LTR	ERVL	LTR57	0.34	**−0.04 (0.898)**	0.36
LTR	ERVL-MaLR	THE1C-int	0.36	0.57	**−0.19**
Satellite	Satellite	HSAT5	0.29 (0.035)	0.67	**−0.36**
Satellite	Satellite	MSR1	0.61	0.28 (0.160)	0.31
snRNA	snRNA	U14	0.82	0.41 (0.204)	0.36 (0.027)
snRNA	snRNA	U17	1.24	0.27 (0.439)	0.93

Repetitive elements upregulated in at least one of the comparisons (LP vs C, NLP vs C or LP vs NLP) accompanied by log_2_ foldchange values from all comparisons are presented in this table. Repetitive element annotations based on RepeatMasker hg19 Library are also listed. Negative log_2_ foldchange values, which denoting downregulation, are written in bold. FDR values are presented in parentheses for log_2_ foldchange values which did not exceed the FDR ≤ 0.01 cut-off. LP – lesional skin; NLP – non-lesional skin; C – control group.

**Figure 1:**
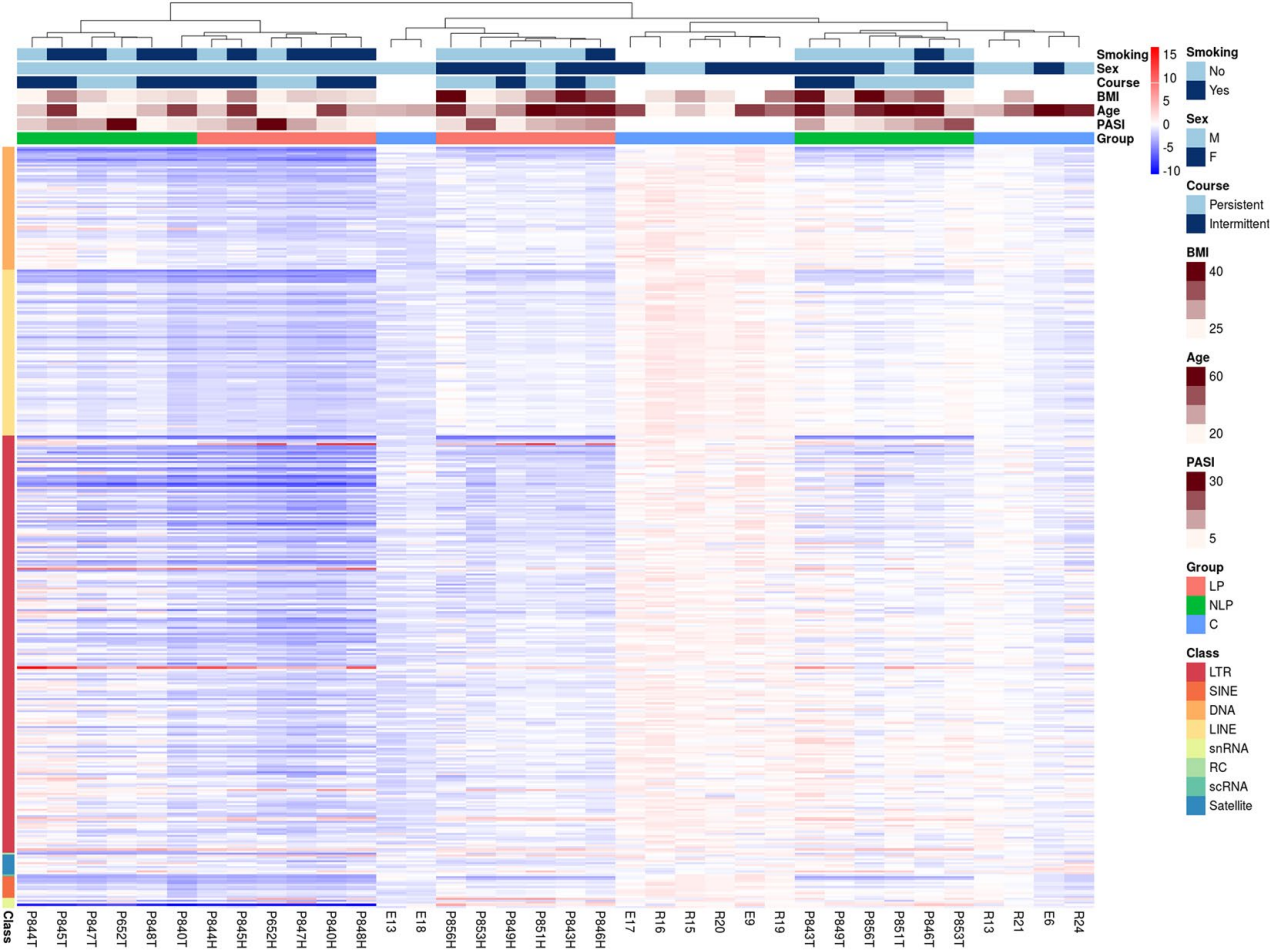
Differentially expressed elements in the lesional, non-lesional and healthy control skin. Expression levels of differentially expressed elements at FDR ≤ 0.01 in lesional skin (LP) vs control group or non-lesional skin (NLP) vs control group (C) comparisons are presented as a heatmap. Samples are clustered based on Euclidean distance calculated from z-score values. For every repetitive element, the mean and standard deviation based on control group CPM values were used for z-score calculation. Selection of patient traits are also presented: Psoriasis Area Severity Index (PASI) score, age, body mass index (BMI), course of the disease, sex and smoking status. Repetitive element classes are color coded. This information was not available for all control group individuals.

The larger part of elements downregulated in NLP compared to C overlapped with elements downregulated in LP compared to C, with the number of elements downregulated in LP being more than four-fold greater than in NLP. This trend was also observed for the upregulated elements (Fig. S6). The graphical representation of the first two principal components and clustering analysis of all elements differentially expressed at FDR ≤ 0.01 in LP vs C or NLP vs C analyses show distinguishable clustering of the expression patterns of the three groups (Figs S7 and S8). Clustering based on z-scores resulted in all three groups being divided into subgroups, while clustering together individuals with greater age or higher body mass index (Fig. 1).

### Differentially expressed elements: pairwise comparison

**Figure 2:**
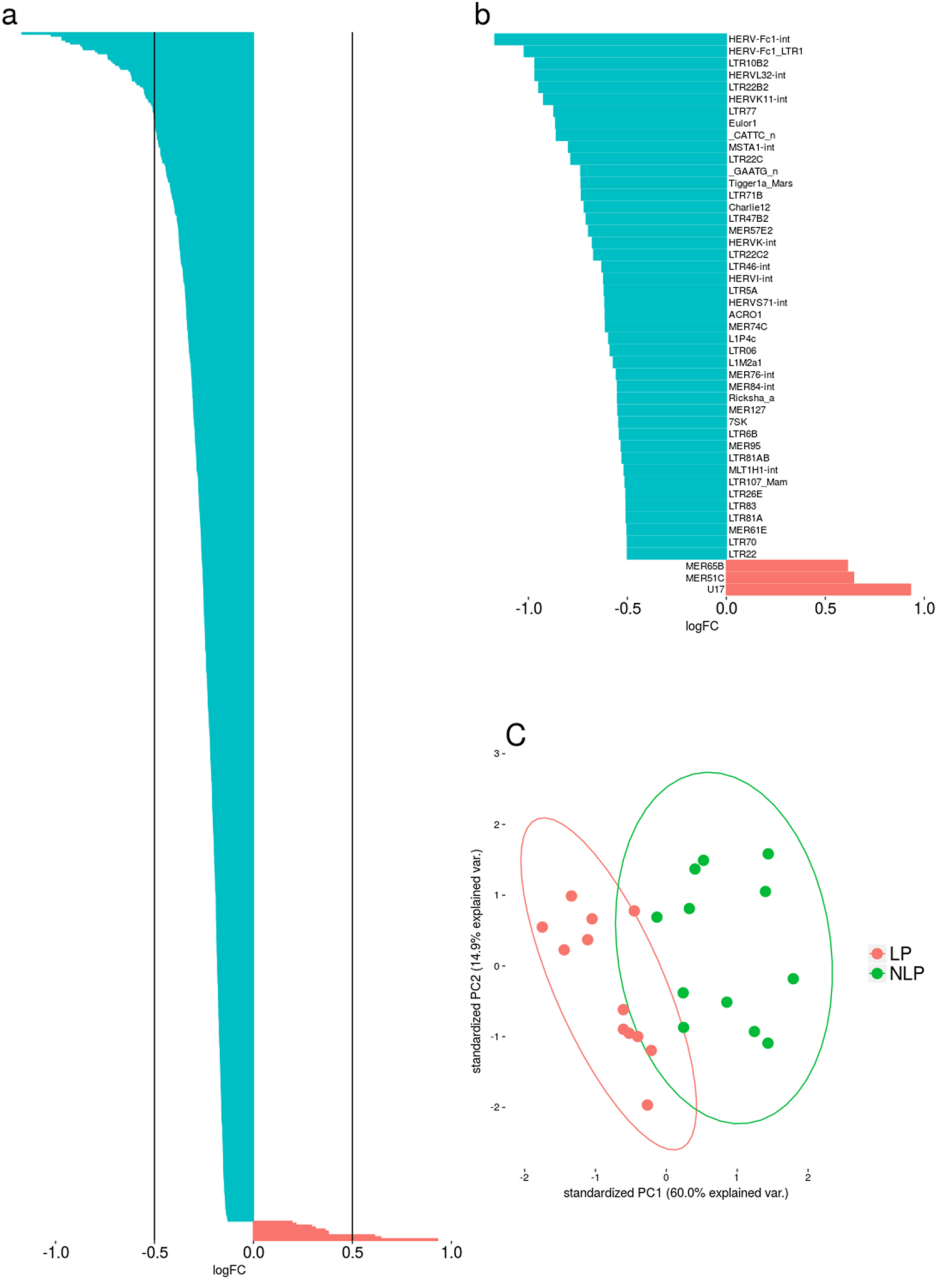
Differentially expressed elements in the lesional and non-lesional skin. (**a**) Distribution of foldchange values as logarithms to the base 2 (log_2_FC) of differentially expressed elements in the pairwise comparison between lesional (LP) and non-lesional (NLP) skin of psoriasis patients. Only differentially expressed elements at FDR ≤ 0.01 are presented. (**b**) Log_2_FC values of differentially expressed elements at FDR ≤ 0.01 in the pairwise comparison limited to log_2_FC ≤ −0.5 or log_2_FC ≥ 0.5 (translating into foldchange greater than 0.71 or 1.41). (**c**) PCA analysis based on counts per million (CPM) values of elements differentially expressed at FDR ≤ 0.01 in the LP vs C and NLP vs C comparisons. Plotted ovals represent the 95% confidence interval.

Pairwise analysis of LP and NLP groups with the edgeR package resulted in 621 DE elements at FDR ≤ 0.01. Elements differentially expressed at a level of log_2_FC ≤ −0.5 or log_2_FC ≥ 0.5 are listed in Table S7. The full listing of DE elements can be found in Table S8. Unlike in LP vs C or NLP vs C comparisons, the number of DE elements from the LTR class was larger than expected (388 out of 621). This is a significant enrichment of LTR elements (p-value = 0.002, Chi-squared test for given probabilities), given the fact that 55% (566 out of 1033) of the expressed elements were LTR elements. In line with trends emerging from the LP vs C and NLP vs C comparisons, 98% of the DE elements were downregulated in the lesional skin tissue compared to visually healthy skin from the same patients (Fig. 2a,b). The few upregulated elements were mostly from the ERV1 family (Table 2). snRNA U17 and satellite repeat MSR1 were also upregulated in the LP group compared to the NLP group. Plotting the first two principal components (Fig. 2c) and clustering based on z-score values (Fig. S7) highlight the differences between LP and NLP groups. The element family level comparison resulted in 36 downregulated families with mostly modest, but statistically significant (FDR ≤ 0.01) fold change values (Table S9).

### Coverage analysis

**Figure 3:**
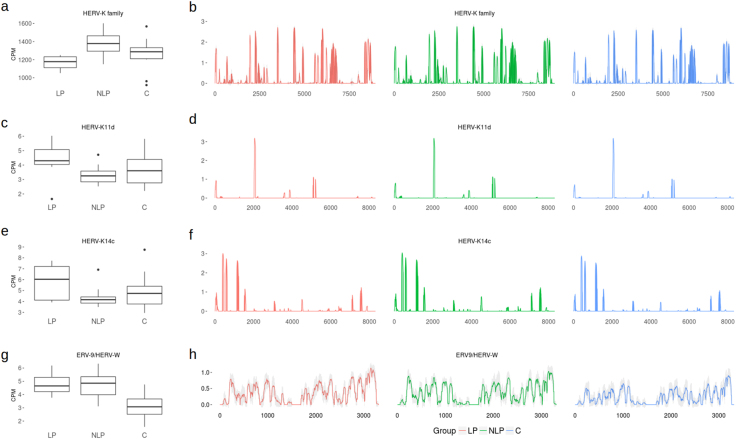
Coverage of HERVs relevant in psoriasis. Comparison of counts per million (CPM) values and sequence coverage of elements regarded relevant in psoriasis. (**a**) CPM values of HERV-K elements summarized at the family level. (**b**) Coverage data from all sequence loci of HERV-K elements displayed along the length of aligned sequences (x-axis) and on y-axis as log_10_ of read depth. (**c**) CPM values of HERV-K11D. (**d**) Coverage data from all HERV-K11D loci displayed along the length of aligned sequences (x-axis) and on y-axis as log_10_ of read depth. (**e**) CPM values of HERV-K14C (**f**) Coverage data from all HERV-K14C loci displayed along the length of aligned sequences (x-axis) and on y-axis as log_10_ of read depth. (**g**) CPM values of the ERV9/HERV-W element^12^ and (**h**) coverage data displayed along the length of the sequences (x-axis) and on y-axis as log_10_ of read depth. The study groups are marked “LP” for lesional skin on psoriasis patients, “NLP” for non-lesional skin of psoriasis patients and “C” for healthy controls.

We performed coverage analysis of the more relevant elements in the context of psoriasis, such as members of the HERV-K family and the novel HERV-W sequence reported by Molès and colleagues^12^. While the HERV-W element and HERV-K sequences showed more uniform coverage (Fig. 3a and d), the specific loci of HERV-K11D and HERV-K14C elements were erratically covered (Fig. 3b,c). This suggests that the latter sequences are not likely expressed in full length.

## Discussion

With on average 27.5% of reads aligning to repetitive element loci, the expression of repetitive elements was prominent in all the 12 lesional skin (LP), 12 non-lesional skin (NLP) and 12 healthy control (C) samples investigated. The repetitive element component of RNA-Seq data has been investigated before, as in a study of postmortem tissue of human frontal cortex, the authors report that 8% of mappable reads originated from repetitive elements^21^. However, the proportion of reads aligning to repetitive elements loci in our data is notably larger, although still in line with ranges previously obtained by Cap Analysis Gene Expression (CAGE) tagging methods^22,23^. Similar to the results obtained by Tyekucheva and colleagues, LINE, SINE, and LTR elements were most abundantly represented in our RNA-sequencing dataset, which was as expected, since these three classes also comprise the larger part of RepeatMasker Library annotations.

The downregulation of more than half of detected LTR elements in the LP and NLP groups hints at active suppression of these elements. This is further supported by the observation that the overall variability of expression levels of repetitive elements is smaller in the LP and NLP groups. The pro-inflammatory environment of psoriasis results in an antiviral state and the increased expression of antiviral proteins, such as the cytidine deaminase APOBEC3G and phosphohydrolase SAMHD1^24^. In our previously published results of differential gene expression analysis based on the same dataset, we observed 1.2- to 3.4-fold upregulation of the aforementioned genes in both LP and NLP samples^16^.

The most prominent mechanisms responsible for suppressing LTRs and other repetitive elements are believed to be methylation and histone modifications^6,25^. It has been reported that the number of hypermethylated regions is increased in the lesional skin of psoriasis patients^26^. In our previously published gene DE analysis results^16^, we observed the increased expression of DNA methyltransferases *DNMT1* and *DNMT3B*, and histone deacetylase *HDAC1*, as well as the decreased expression of histone acetyltransferase encoding genes (*EP300, CREBBP* and *SIRT1*) in the LP and NLP samples compared to healthy controls. The changes in expression levels of the aforementioned antiviral elements and chromatin modifying enzymes are in concordance with the gradually reduced expression of downregulated repetitive elements in the NLP and LP samples, supporting the involvement of these mechanisms in the observed downregulation of repetitive elements.

HERV-K, as the most recently active HERV family in the human genome^27^, has lately been actively investigated in terms of autoimmune diseases^28,29^. We observed that the DE elements from the HERV-K family were downregulated in the LP and NLP tissues compared to controls, as previously reported by Gupta and colleagues^15^. In the LP group, the downregulation was statistically significant at the element family level as well.

The background of suppressed repetitive elements raises the intriguing question of why some of the elements are upregulated in LP and NLP tissues. The activation of LTR elements can be caused by *de novo* loss of epigenetic silencing at a given locus^30^. Interestingly, Roberson and colleagues have reported that the loci of genes often upregulated in psoriatic lesion are hypomethylated compared to healthy skin^31^. This could potentially affect the transcription of nearby LTR elements as well, leading us to consider the possibility of repetitive element expression being induced by changes in the gene expression profile inherent to the pro-inflammatory environment in psoriasis. Once expressed, cytosolic retroviral nucleic acids and proteins can be detected by TLRs as PAMPs or DAMPs^9,14^. Thus, the increased expression of HERVs could, in turn, contribute to the already established pro-inflammatory environment.

We detected minor upregulation (1.20 to 1.34-fold) of HERV-W (HERV-17) elements in the LP and NLP tissues compared to controls. Pro-inflammatory transcription factors have been shown to have an inducing effect on HERV-W expression^32^. Increased expression of HERV-W elements and a novel HERV variant in psoriatic skin has been previously reported by Molès and colleagues^12^. In line with these results, we report the increased expression of the aforementioned HERV variant in the LP and NLP tissues. However, differing from the findings of Molès and colleagues, who reported the novel HERV variant being mostly undetectable in healthy skin, using the consensus sequence provided, we observed the expression of the HERV-W/HERV9 variant in the healthy skin of control group subjects as well.

In recent years, it has been demonstrated that the cytosolic sensing of excess amounts of extracellular DNA (cyDNA) can be mediated by antimicrobial peptides such as LL37. This can lead to a pro-inflammatory state, involving the activation of TLR pathways^33,34^. A recent study reports the presence cytosolic RNA:DNA duplexes in psoriatic lesions generated by endogenous reverse transcriptases, which can contribute to exceeding the tolerance for cyDNAs in keratinocytes and therefore play a role in the disease initiation^35^. We report a ~1.4-fold upregulation of two members of HERV-K family (HERV-K11d and HERV-K14c) encoding reverse transcriptase genes in lesional skin compared to unlesional skin of psoriasis patients. However, the results of this study suggest that these sequences are not likely expressed in full length.

In conclusion, the pro-inflammatory environment seems to result in a general suppression of repetitive elements. Conversely, against this background of suppression, a small subset of HERVs, some of which previously described in the context of psoriasis and other autoimmune diseases, are upregulated. The described differential expression of repetitive elements adds to the evidence of their potential role in the immunopathogenesis of psoriasis. This study, as well as the previous investigations, suggest a complex interplay between autoimmune processes and repetitive element expression which can not be determined from expression data alone, but calls for a integrated approach involving also inquiries into chromatin modifications, locus-specific effects of *cis*- and *trans*-regulatory elements and immunoreactivity of potentially functional endogenous retroviral proteins.

## Materials and Methods

### Patients and Controls

The protocols and informed consent forms used in this study were approved by the Ethics Review Committee on Human Research of the University of Tartu. The participants signed an informed consent form and all the study procedures conformed to the relevant regulatory standards. The patients (*N* = 12) were affected by plaque psoriasis, with PASI scores ranging from 4 to 32, and recruited in the Department of Dermatology of the Tartu University Hospital. The age- and sex-matched control group subjects (*N* = 12) did not show any inflammatory skin conditions and were without a positive family history of psoriasis. Both groups consisted of unrelated Caucasians living in Estonia. For a more detailed description of individuals enrolled in this study, please refer to Keerman *et al*.^16^.

### RNA sequencing

Total RNA was extracted from skin samples of patients and control group subjects obtained by 4 mm punch biopsy. From patients, both lesional (LP) and nonlesional (NLP) tissue were sampled. This resulted in a total of 36 skin biopsy samples. The cDNA libraries were sequenced on the SOLiD 5500 W platform using 75 bp single-end chemistry (Life Technologies Corp., Carlsbad, CA, USA). For the in-depth description of RNA extraction, library preparation and sequencing, please see Keerman *et al*.^16^.

### Read alignment and quantification

Raw color-space reads were filtered for rRNA, active tRNA, and SOLiD adaptor sequences. For quantification purposes, the remaining reads were aligned to the hg19 reference genome while allowing multi-mapping in order to detect reads aligning to possible repeat sequences. No mapping quality cut-off was set in this stage to ensure that secondary alignments were reported in the BAM files. LifeScope software (Life Technologies, Ltd) with recommended settings, designed for color-space read alignment and analysis, was used to perform both mapping steps.

In order to connect color-space mapping with the RepEnrich pipeline^18^, BAM files resulting from mapping to the hg19 were parsed using samtools^36^ and in-house perl scripts to separate uniquely and non-uniquely mapping reads. For uniquely mapping reads, only alignments with MAPQ ≥ 10 (in Phred scale) were retained. For multi-mapping reads, the base-space sequence was inferred from the longest alignment and these reads were converted to FASTQ format.

RepEnrich pipeline with default parameters was applied to obtain read counts of repetitive elements, excluding simple repeats. The alignments were quantified at repeat class, family and subfamily level. Repeat subfamilies are a collection of highly similar sequences representing all known instances of a given repetitive element copies in the hg19 genome build annotated in the RepeatMasker Library. The repetitive element subfamilies as quantified units are referred to as *repetitive elements* or simply as *elements* by the authors. RepEnrich pipeline applies different quantification strategies to uniquely and multi-mapping reads in order to more accurately infer read counts, which reflect the true abundance of expressed repetitive elements.

In order to investigate the coverage of individual elements, we extracted the alignment data for all genomic instances of a given repetitive element annotated in the RepeatMasker hg19 Library. Maintaining the read alignment information, all the genomic instances were aligned using a local version of Clustal Omega v1.2.4^37^ with default parameters. The contribution of multi-mapping reads to the coverage of a given position in the sequence was normalized against the number of alternative alignments for the read (1/*n*, where *n* is the number of alternative alignments), similar to the approach used in the RepEnrich pipeline.

### Analysis of differentially expressed repetitive elements

The edgeR package^38^ in R was used for the analysis of differentially expressed (DE) elements at both element and element family level. The normalization factors, which edgeR uses to normalize against RNA composition were calculated based on both repetitive element counts and counts of the coding genes to account for possible composition biases deriving from the coding part of the library as well. We used the paired sample model design for comparing LP and NLP samples as they originated from the same patient. In the groupwise comparisons of LP and NLP groups with the control group, age was used as a covariate. The edgeR quasi-likelihood pipeline was used for all analyses.

### Statistical calculations and data visualization

The Chi-squared test and the Mann-Whitney U test were conducted using R core functions^39^. Ggpubr package^40^ was used for correlation coefficient calculation and visualization. The mean and standard deviation for z-score calculations were derived from the mean control group counts per million (CPM) values for every repetitive element subfamily. The total number of mapped reads in millions was used as the denominator for calculating CPM values. For reads per kilobase of transcript per million mapped reads (RPKM) calculations, the mean locus length over all genomic instances for a given repetitive element subfamily was used as the transcript length. Principle component analysis (PCA) was conducted with prcomp method of the stats core package in R^39^ and the results were visualized using ggbiplot package^41^. The pheatmap package^42^ was used for heatmap vizualisation and unsupervised hierarchical clustering based on Euclidean distance. The barplots of differentially expressed elements were constructed using ggplot2^43^.

### Data availability

The datasets analysed during the current study are available in the NCBI Gene Expression Omnibus (NCBI-GEO) repository. The accession number for dataset is GSE66511 (https://www.ncbi.nlm.nih.gov/geo/query/acc.cgi?acc = GSE66511). All data generated during this study are included in this published article (and its Supplementary Information files).

## Electronic supplementary material

Supplementary figures and tables

Counts data
