# Supplementary material for: Transcriptional landscape of human endogenous retroviruses (HERVs) and other repetitive elements in psoriatic skin
Source: Sci Rep. 2018 Mar 12;8:4358. doi: 10.1038/s41598-018-22734-7 (PMC5847543; doi:10.1038/s41598-018-22734-7)
Supplement: Supplementary file 1 — Supplementary figures and tables [file 41598_2018_22734_MOESM1_ESM.pdf]

Transcriptional landscape of human endogenous retroviruses (HERVs) and other repetitive elements in psoriatic skin

Freddy Lättekivi[1], Sulev Kõks[1,4], Maris Keermann[2,3], Ene Reimann[1], Ele Prans[1], Kristi Abram[2,3], Helgi Silm[2,3], Gea Kõks[1] and Külli Kingo[2,3]

[1]Department of Pathophysiology, University of Tartu, Tartu, Estonia

[2]Department of Dermatology, University of Tartu, Tartu, Estonia

[3]Clinic of Dermatology, Tartu University Hospital, Tartu, Estonia

[4]Department of Reproductive Biology, Estonian University of Life Sciences, Tartu, Estonia

SUPPLEMENTARY FIGURES AND TABLES

Table of Contents

Figure S1: Sequencing and read alignment results.....3
Figure S2: RPKM values of repetitive elements.....4
Figure S3: Correlation between RPKM values and number of element loci.....5
Figure S4: Variability of repetitive element expression levels in study groups.....6
Figure S6: Venn diagram of differentially expressed elements.....8
Figure S7: Differentially expressed elements in the lesional, non-lesional and healthy control skin.....9
Figure S8: PCA analysis of differentially expressed elements in the lesional and non-lesional skin.....10
Table S1: Results of the LP vs C comparison with edgeR. Elements differentially expressed at FDR ≤ 0.01 limited to $log_2FC \leq -0.5$ or $log_2FC \geq 0.5$.....11
Table S2: Results of the NLP vs C comparison with edgeR. Elements differentially expressed at FDR ≤ 0.01.....14
Table S3: Results of the LP vs C comparison with edgeR. List of all elements differentially expressed at FDR ≤ 0.01 limited to $log_2FC \leq -0.5$ or $log_2FC \geq 0.5$.....15
Table S4: Results of the NLP vs C comparison with edgeR. List of all elements differentially expressed at FDR ≤ 0.01.....24
Table S5: Results of the family-level LP vs C comparison with edgeR. List of all families differentially expressed at FDR ≤ 0.01.....26
Table S6: Results of the family-level NLP vs C comparison with edgeR. List of all families differentially expressed at FDR ≤ 0.01.....27
Table S7: Results of the pairwise comparison with edgeR. List of all elements differentially expressed at FDR ≤ 0.01 limited to $log_2FC \leq -0.5$ or $log_2FC \geq 0.5$.....28
Table S8: Results of the pairwise comparison with edgeR. List of all elements differentially expressed at FDR ≤ 0.01.....30
Table S9: Results of the family-level pairwise comparison with edgeR. List of all families differentially expressed at FDR ≤ 0.01.....43

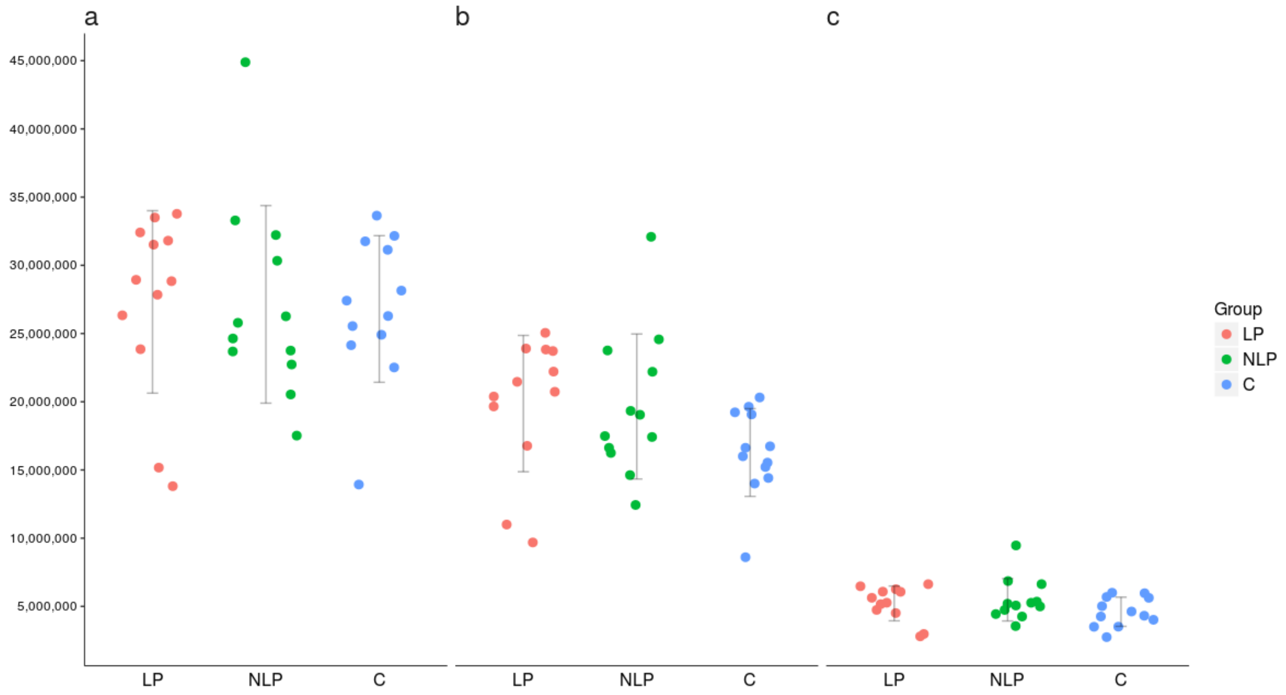

Figure S1: Sequencing and read alignment results. (a) Total number of sequenced reads per sample. **(b)** Amount of reads per sample mapped to the hg19 reference genome using LifeScope color-space mapping module with no MAPQ cut-off. **(c)** Out of total mapped reads, number of reads per sample mapping to repetitive element loci. Standard error bars (mean +/- SD) are plotted for all three groups alongside individual results.

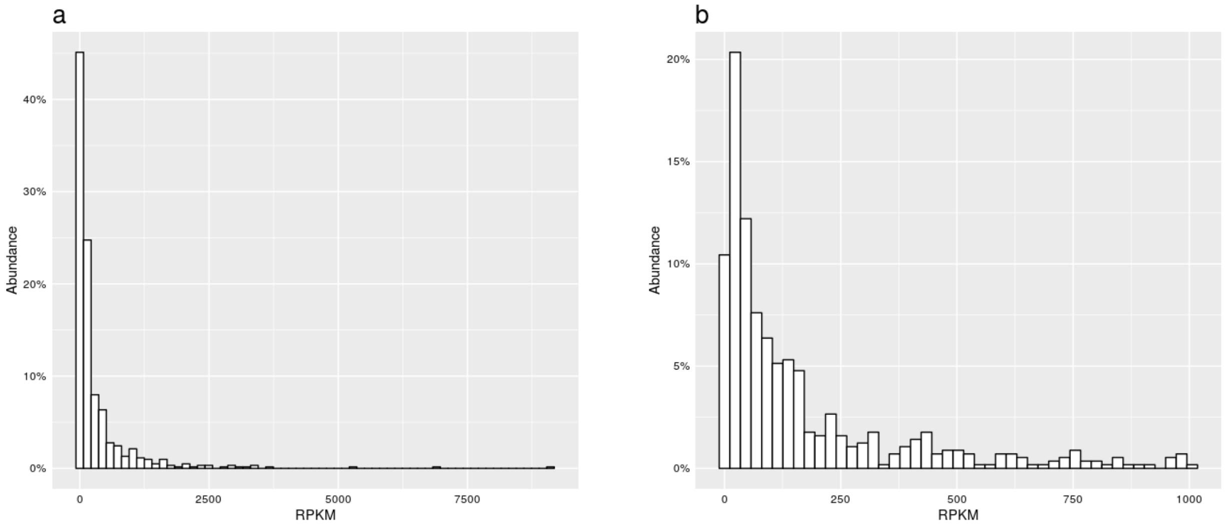

Figure S2: RPKM values of repetitive elements. (b) Distribution of RPKM (Reads Per Kilobase of transcript per Million mapped reads) values of differentially expressed elements at FDR ≤ 0.01 (Two elements with more extreme values, AluSx and AluSp with RPKMs 40 768 and 14 565, respectively, are left out of this graph); **(b)** distribution of RPKM values of differentially expressed elements at FDR ≤ 0.01 limited to RPKM ≤ 1000.

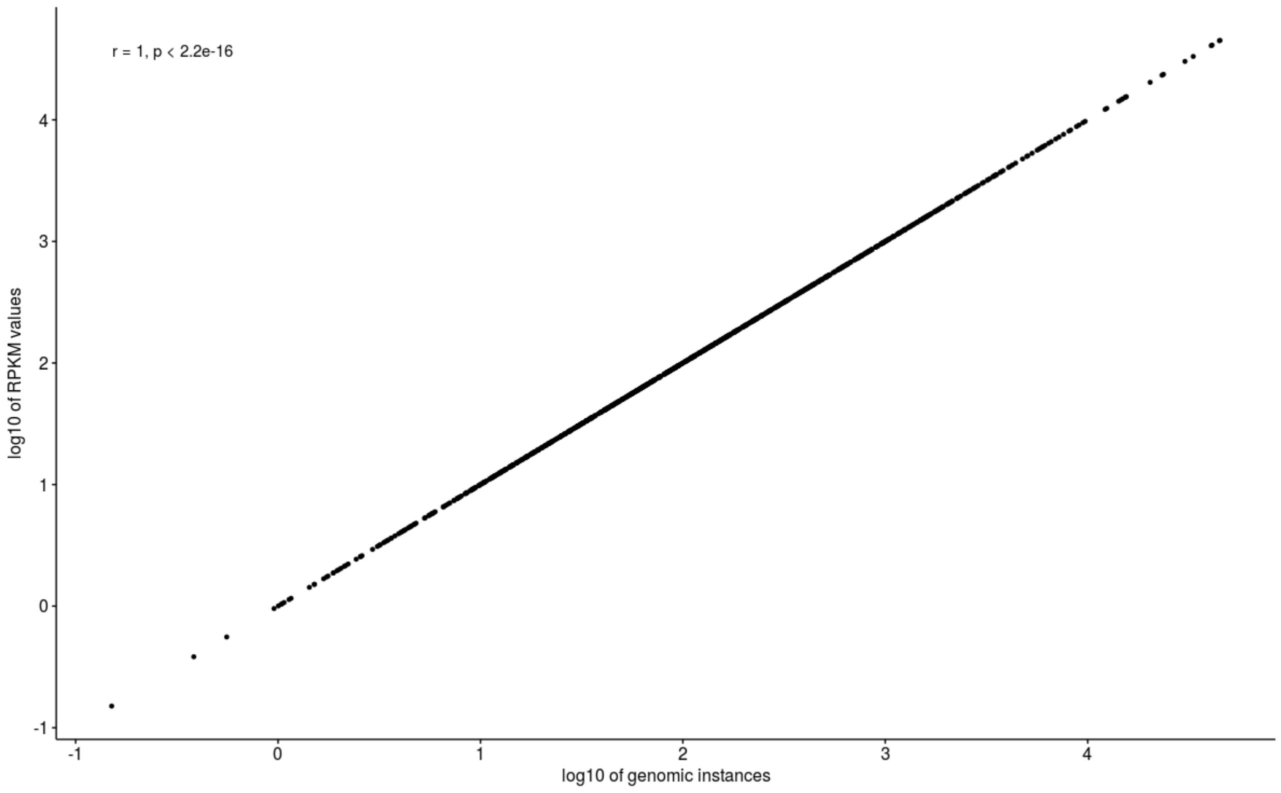

Figure S3: Correlation between RPKM values and number of element loci . The number of genomic instances for all repetitive elements expressed at RPKM ≥ 1 is plotted along the x-axis with RPKM (Reads Per Kilobase of transcript per Million mapped reads) values on the y-axis. Both axes are $\log_{10}$-transformed. The correlation coefficient calculated is Pearson's *r*.

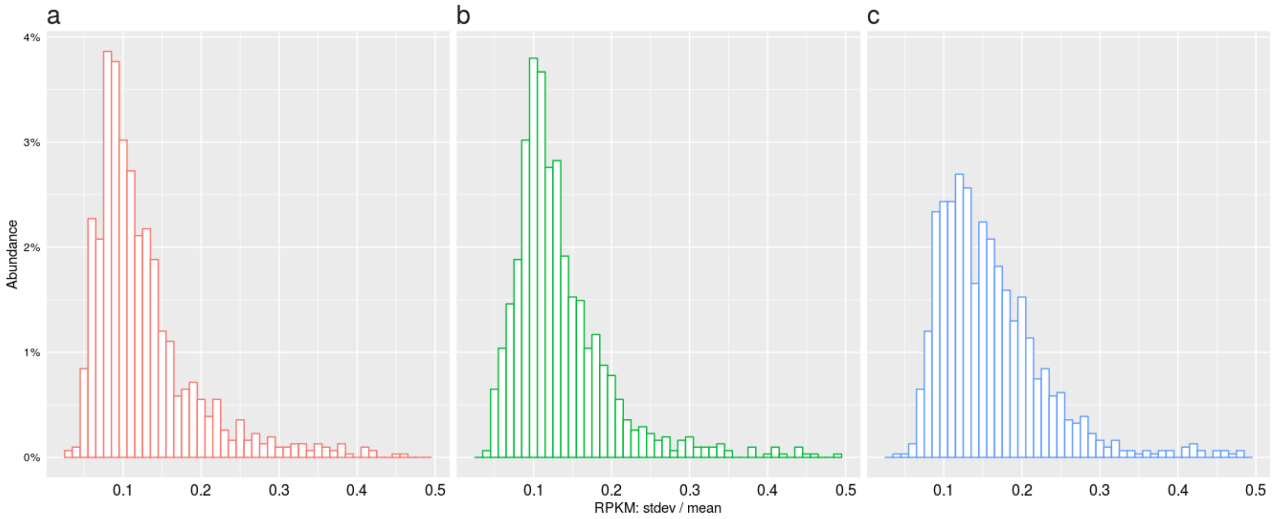

Figure S4: Variability of repetitive element expression levels in study groups. The figure displays distribution of normalized standard deviations (SD divided by mean) of RPKM (Reads Per Kilobase of transcript per Million mapped reads) values for every element in all three study groups, showing the variability of repetitive element expression in lesional skin (LP) group **(a)**, non-lesional skin (NLP) group **(b)** and healthy control (C) group **(c)**.

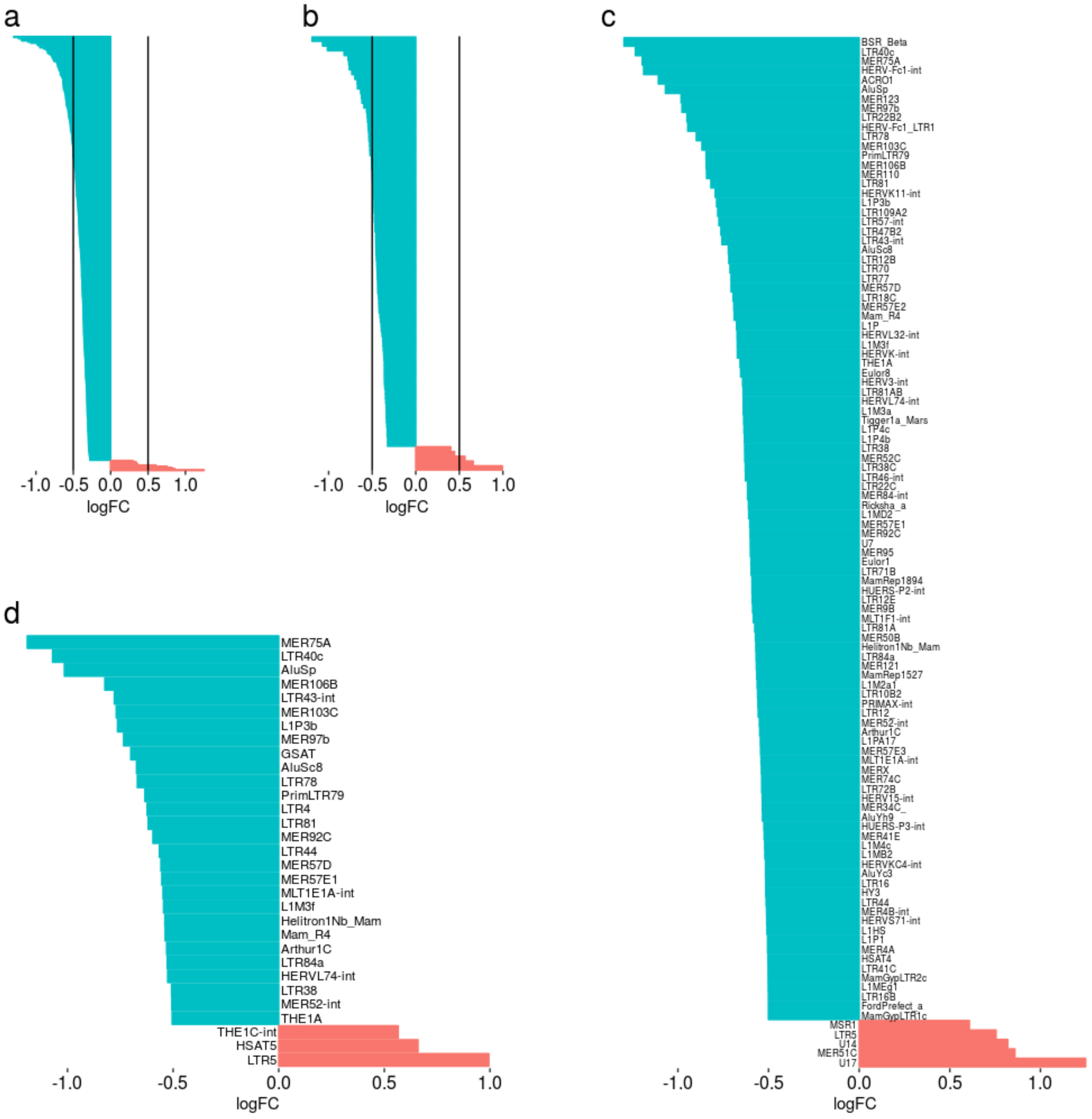

Figure S5: The proportions of upregulated and downregulated repetitive elements.(a) Distribution of foldchange values as logarithms to the base 2 (log_2FC) of differentially expressed elements in the groupwise comparison between non-lesional psoriatic (NLP) and healthy control (C) skin. Only differentially expressed elements at FDR ≤ 0.01 are presented. **(b)** Distribution of foldchange values as logarithms to the base 2 (log_2FC) of differentially expressed elements in the pairwise comparison between lesional psoriatic (LP) and healthy control (C) skin. Only differentially expressed elements at FDR ≤ 0.01 are presented. **(c)** Log_2FC values of differentially expressed elements at FDR ≤ 0.01 in the comparison between lesional psoriatic (LP) and healthy control (C) skin limited to log_2FC ≤ -0.5 or log_2FC ≥ 0.5 (translating into foldchange greater than 0.71 or 1.41). **(d)** Log_2FC values of differentially expressed elements at FDR ≤ 0.01 in the comparison between non-lesional (NLP) and healthy control skin (C) limited to log_2FC ≤ -0.5 or log_2FC ≥ 0.5.

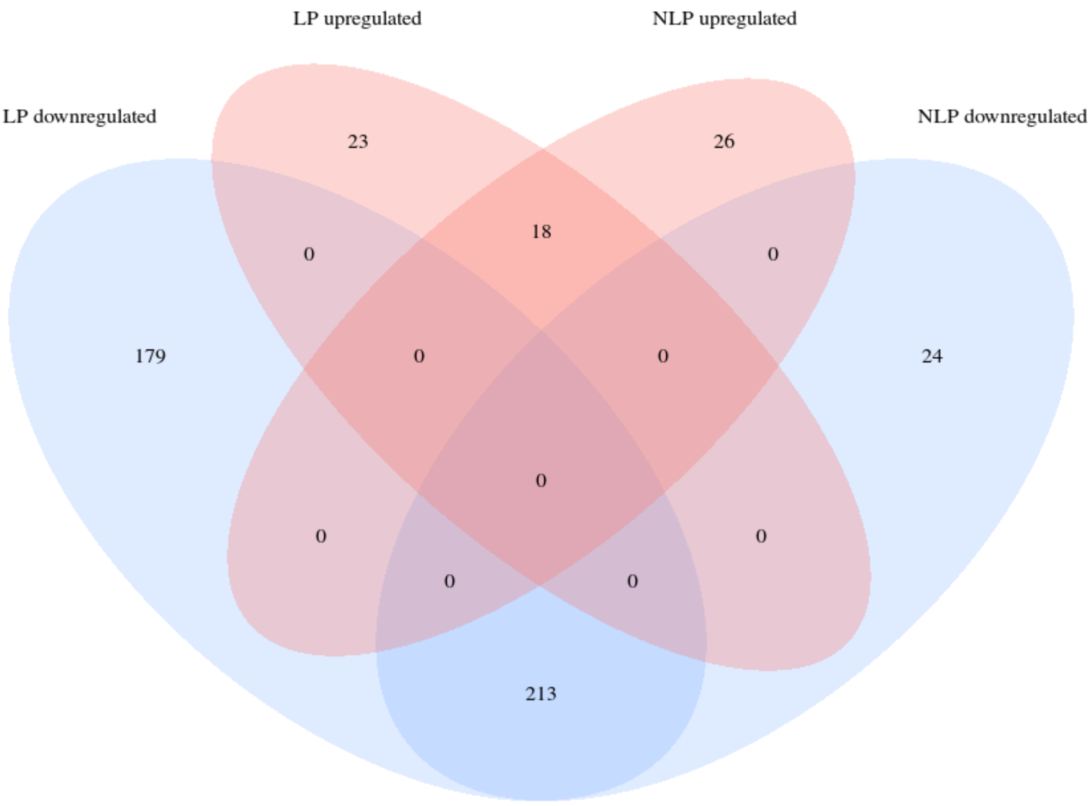

Figure S6: Venn diagram of differentially expressed elements. The presented elements were differentially expressed at FDR ≤ 0.01 in the lesional skin (LP) vs control (C) and non-lesional skin (NLP) vs control (C) comparisons

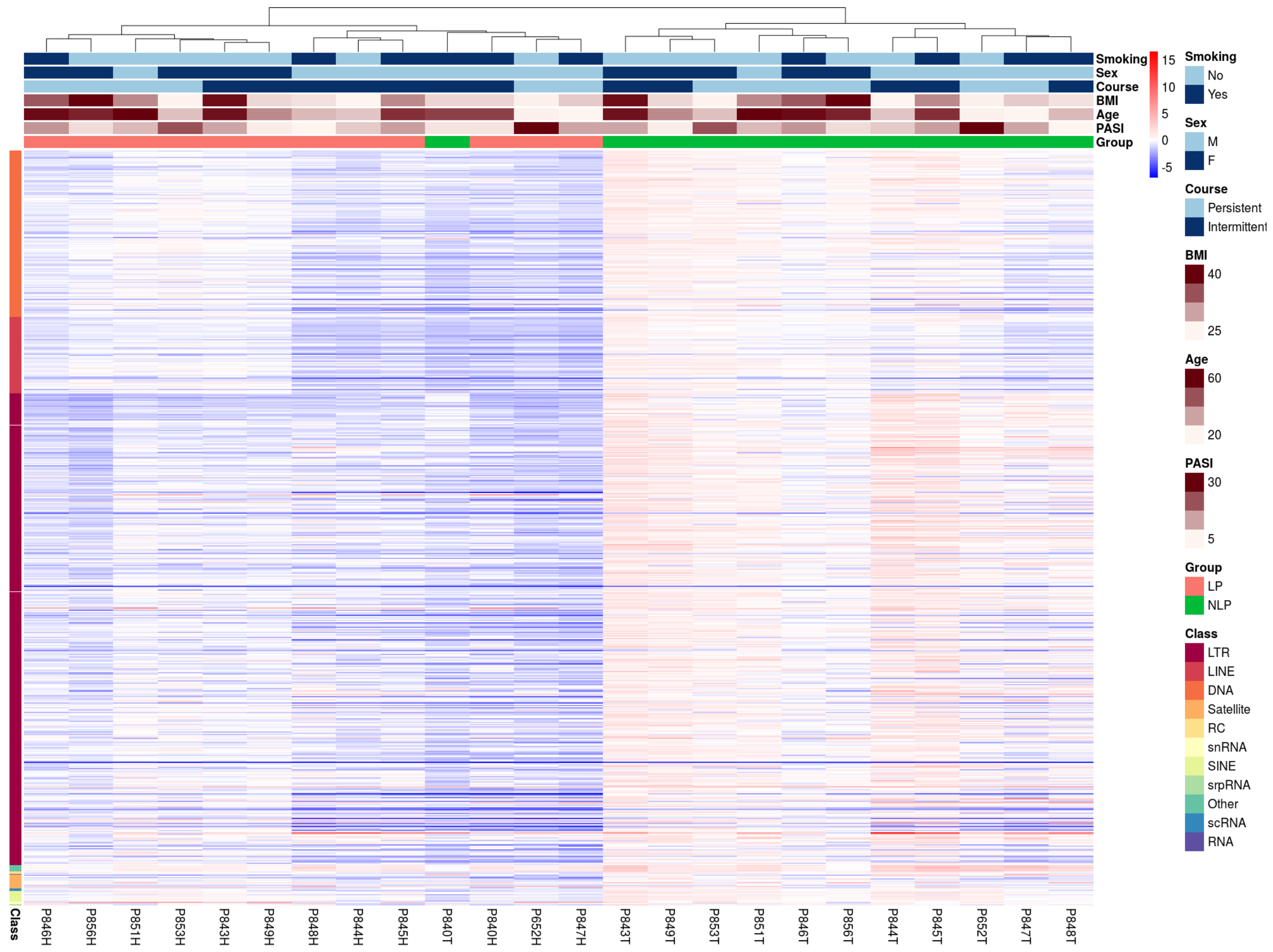

Figure S7: **Differentially expressed elements in the lesional, non-lesional and healthy control skin. Differentially expressed elements in the lesional and non-lesional skin.** Expression levels of differentially expressed elements at FDR ≤ 0.01 in lesional skin (LP) vs non-lesional skin (NLP) pairwise comparison are presented as a heatmap. Samples are clustered based on Euclidean distance calculated from z-score values. For every repetitive element, the mean and standard deviation based on control group CPM values were used for z-score calculation. Selection of patient traits are also presented: PASI score, age, BMI, course of the disease, sex and smoking status. Repetitive element classes are color coded.

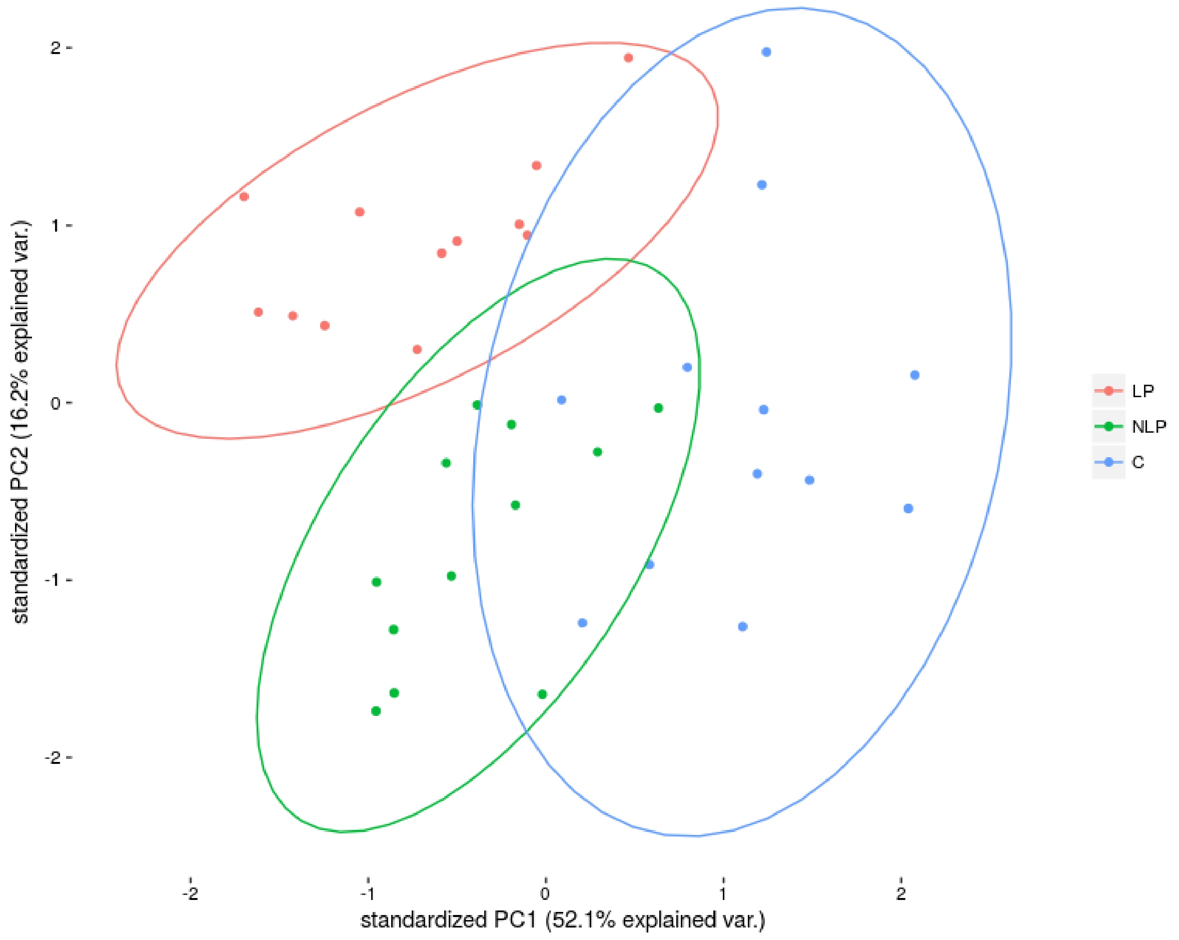

Figure S8: PCA analysis of differentially expressed elements in the lesional and non-lesional skin. PCA analysis is based on counts per million (CPM) values of elements differentially expressed at FDR ≤ 0.01 in the LP vs C or NLP vs C comparisons. Plotted ovals represent the 95% confidence interval.

Table S1: Results of the LP vs C comparison with edgeR. Elements differentially expressed at FDR ≤ 0.01 with $log_2FC \leq -0.5$ or $log_2FC \geq 0.5$ (translating into foldchange difference greater than 0.71 or 1.41).

**Class**	**Family**	**Subfamily**	**log_2FC**	**p-value**	**FDR**
DNA	DNA	Eulor1	-0.61	9.47E-04	3.07E-03
DNA	DNA	MER123	0.82	2.85E-06	2.71E-05
DNA	hAT	MamRep1894	1.24	1.02E-08	2.51E-07
DNA	hAT-Blackjack	MER94B	-1.07	4.95E-06	4.30E-05
DNA	hAT-Charlie	MER103C	-0.73	2.69E-19	4.63E-17
DNA	hAT-Charlie	MER106B	-0.55	1.49E-19	3.08E-17
DNA	hAT-Tip100	Arthur1C	-0.55	1.96E-08	4.39E-07
DNA	hAT-Tip100	FordPrefect_a	-0.54	8.22E-08	1.36E-06
DNA	hAT-Tip100	MER97b	-1.17	2.60E-15	2.99E-13
DNA	MULE-MuDR	Ricksha_a	-1.12	5.05E-05	2.90E-04
DNA	PiggyBac	MER75A	-0.66	2.11E-21	7.25E-19
DNA	TcMar-Tigger	MERX	-0.52	4.11E-07	5.31E-06
DNA	TcMar-Tigger	Tigger1a_Mars	-0.51	1.26E-04	6.10E-04
DNA	TcMar?	Eulor8	0.61	2.68E-03	7.32E-03
DNA	TcMar?	MER121	-0.61	2.11E-08	4.55E-07
LINE	Dong-R4	Mam_R4	-1.25	2.36E-11	9.74E-10
LINE	L1	L1HS	-1.2	8.31E-08	1.36E-06
LINE	L1	L1M2a1	-0.96	3.37E-07	4.58E-06
LINE	L1	L1M3a	-0.92	8.45E-12	3.97E-10
LINE	L1	L1M3f	-0.9	1.81E-12	1.10E-10
LINE	L1	L1M4c	-0.89	1.68E-07	2.64E-06
LINE	L1	L1MB2	-0.88	1.53E-08	3.50E-07
LINE	L1	L1MD2	-0.85	1.33E-10	4.91E-09
LINE	L1	L1MEg1	-0.84	1.85E-07	2.82E-06
LINE	L1	L1P	-0.81	1.42E-06	1.50E-05
LINE	L1	L1P1	-0.8	2.49E-07	3.52E-06
LINE	L1	L1P2	-0.78	2.11E-07	3.03E-06
LINE	L1	L1P3b	-0.77	1.14E-13	7.84E-12
LINE	L1	L1P4b	-0.76	4.36E-07	5.49E-06
LINE	L1	L1P4c	-0.74	2.72E-03	7.38E-03
LINE	L1	L1PA17	-0.72	5.99E-09	1.65E-07
LINE	L1	L1PBb	-0.72	1.91E-07	2.85E-06
LTR	ERV1	HERV-Fc1_LTR1	-0.71	5.22E-05	2.98E-04
LTR	ERV1	HERV-Fc1-int	-0.71	3.68E-19	5.42E-17
LTR	ERV1	HERV15-int	-0.69	3.70E-06	3.29E-05
LTR	ERV1	HERV3-int	-0.69	1.27E-08	3.06E-07
LTR	ERV1	HERVS71-int	-0.68	2.53E-07	3.53E-06
LTR	ERV1	HUERS-P2-int	-0.68	3.41E-08	7.04E-07
LTR	ERV1	HUERS-P3-int	-0.67	7.90E-08	1.36E-06
LTR	ERV1	LTR109A2	-0.67	2.05E-03	5.90E-03
LTR	ERV1	LTR10B2	-0.67	6.99E-07	8.20E-06
LTR	ERV1	LTR12_	-0.66	4.26E-08	8.31E-07
LTR	ERV1	LTR12B	-0.65	2.77E-10	9.85E-09
LTR	ERV1	LTR12E	-0.65	5.27E-08	9.71E-07
LTR	ERV1	LTR38	-0.64	5.97E-09	1.65E-07

LTR	ERV1	LTR38C	-0.64	3.90E-08	7.89E-07
LTR	ERV1	LTR4	-0.63	8.18E-08	1.36E-06
LTR	ERV1	LTR43-int	-0.63	8.94E-14	6.60E-12
LTR	ERV1	LTR44	-0.62	1.98E-06	2.02E-05
LTR	ERV1	LTR46-int	-0.62	7.28E-09	1.83E-07
LTR	ERV1	LTR70	-0.62	2.25E-11	9.69E-10
LTR	ERV1	LTR71B	-0.61	2.02E-07	2.98E-06
LTR	ERV1	LTR72B	-0.61	1.10E-05	8.80E-05
LTR	ERV1	LTR77	-0.6	6.35E-08	1.13E-06
LTR	ERV1	LTR78	-0.6	6.80E-21	1.76E-18
LTR	ERV1	MER110	-0.6	1.97E-14	1.85E-12
LTR	ERV1	MER34A	-0.59	2.63E-03	7.30E-03
LTR	ERV1	MER34C_	-0.59	4.56E-08	8.57E-07
LTR	ERV1	MER41E	-0.59	1.59E-06	1.65E-05
LTR	ERV1	MER4A	-0.59	1.42E-07	2.25E-06
LTR	ERV1	MER4B-int	-0.58	6.45E-08	1.13E-06
LTR	ERV1	MER50B	-0.58	1.33E-08	3.12E-07
LTR	ERV1	MER51C	-0.58	1.81E-14	1.85E-12
LTR	ERV1	MER52-int	-0.57	3.97E-09	1.21E-07
LTR	ERV1	MER52C	-0.57	3.13E-09	9.79E-08
LTR	ERV1	MER57D	-0.55	2.64E-12	1.36E-10
LTR	ERV1	MER57E1	-0.55	1.19E-10	4.60E-09
LTR	ERV1	MER57E2	-0.54	3.12E-06	2.93E-05
LTR	ERV1	MER57E3	-0.54	3.15E-07	4.33E-06
LTR	ERV1	MER67A	-0.54	9.16E-07	1.04E-05
LTR	ERV1	MER84-int	-0.54	7.89E-07	9.06E-06
LTR	ERV1	MER92C	-0.54	6.59E-09	1.74E-07
LTR	ERV1	MER95	-0.54	8.54E-06	7.05E-05
LTR	ERV1	PRIMAX-int	-0.54	8.71E-04	2.85E-03
LTR	ERV1	PrimLTR79	-0.53	3.07E-14	2.65E-12
LTR	ERVK	HERVK-int	-0.53	1.44E-11	6.47E-10
LTR	ERVK	HERVK11-int	-0.53	2.06E-15	2.66E-13
LTR	ERVK	HERVKC4-int	-0.52	1.13E-04	5.58E-04
LTR	ERVK	LTR22B2	-0.52	6.76E-09	1.74E-07
LTR	ERVK	LTR22C	-0.52	1.75E-03	5.15E-03
LTR	ERVK	LTR5	-0.52	4.73E-05	2.76E-04
LTR	ERVK	MER9B	-0.51	2.65E-04	1.09E-03
LTR	ERVL	HERVL32-int	-0.51	2.08E-08	4.55E-07
LTR	ERVL	HERVL74-int	-0.51	1.20E-10	4.60E-09
LTR	ERVL	LTR16	-0.51	3.08E-08	6.50E-07
LTR	ERVL	LTR16B	0.76	1.75E-07	2.70E-06
LTR	ERVL	LTR18C	0.85	4.23E-05	2.54E-04
LTR	ERVL	LTR40c	-0.83	3.51E-37	3.63E-34
LTR	ERVL	LTR41C	-0.71	1.11E-06	1.20E-05
LTR	ERVL	LTR47B2	-0.69	7.73E-10	2.66E-08
LTR	ERVL	LTR53	-0.69	3.37E-06	3.08E-05
LTR	ERVL	LTR57-int	-0.66	7.57E-12	3.72E-10
LTR	ERVL	LTR83	-0.65	2.47E-06	2.46E-05
LTR	ERVL	LTR84a	-0.64	6.06E-09	1.65E-07
LTR	ERVL	MER74C	-0.62	3.24E-05	2.10E-04

LTR	ERVL-MaLR	MLT1E1A-int	-0.57	2.07E-07	3.01E-06
LTR	ERVL-MaLR	MLT1F1-int	-0.56	2.70E-09	8.71E-08
LTR	ERVL-MaLR	THE1A	-0.55	2.41E-12	1.31E-10
LTR	Gypsy	LTR81	-0.52	2.29E-12	1.31E-10
LTR	Gypsy	LTR81A	-0.52	5.85E-08	1.06E-06
LTR	Gypsy	LTR81AB	-0.52	1.72E-05	1.26E-04
LTR	Gypsy	MamGypLTR1c	-0.51	4.51E-07	5.62E-06
LTR	Gypsy	MamGypLTR2c	-0.51	9.95E-08	1.61E-06
LTR	LTR	MamRep1527	-0.5	5.31E-09	1.57E-07
RC	Helitron	Helitron1Nb_Mam	-1.26	1.15E-09	3.84E-08
Satellite	acro	ACRO1	-1.01	1.94E-04	8.45E-04
Satellite	centr	ALR_Alpha	-1	3.84E-03	9.61E-03
Satellite	centr	HSAT4	-0.89	1.34E-05	1.01E-04
Satellite	Satellite	BSR_Beta	-0.87	9.05E-05	4.70E-04
Satellite	Satellite	CER	-0.66	2.75E-06	2.65E-05
Satellite	Satellite	MSR1	-0.64	1.08E-06	1.19E-05
scRNA	scRNA	HY3	-0.62	4.49E-08	8.57E-07
SINE	Alu	AluSc8	-0.62	8.31E-14	6.60E-12
SINE	Alu	AluSp	-0.6	1.37E-27	7.06E-25
SINE	Alu	AluYc3	-0.59	4.03E-08	8.01E-07
SINE	Alu	AluYh9	-0.58	3.51E-07	4.68E-06
snRNA	snRNA	U14	-0.56	3.01E-05	1.99E-04
snRNA	snRNA	U17	-0.54	1.54E-12	9.96E-11
snRNA	snRNA	U7	-0.51	7.89E-07	9.06E-06

Table S2: Results of the NLP vs C comparison with edgeR. Elements differentially expressed at FDR ≤ 0.01 with $log_2FC \leq -0.5$ or $log_2FC \geq 0.5$ (translating into foldchange difference greater than 0.71 or 1.41).

Class	Family	Subfamily	log_2FC	p-value	FDR
DNA	hAT-Charlie	MER103C	-1.01	8.45E-16	1.74E-13
DNA	hAT-Charlie	MER106B	-0.67	1.46E-17	3.77E-15
DNA	hAT-Tip100	Arthur1C	0.66	3.92E-07	1.74E-05
DNA	hAT-Tip100	MER97b	-0.7	1.31E-08	1.23E-06
DNA	PiggyBac	MER75A	-0.54	1.24E-18	4.27E-16
LINE	Dong-R4	Mam_R4	-1.07	4.40E-07	1.74E-05
LINE	L1	L1M3f	-0.78	1.86E-08	1.60E-06
LINE	L1	L1P3b	-0.67	1.93E-11	2.21E-09
LTR	ERV1	LTR38	0.57	1.76E-05	3.64E-04
LTR	ERV1	LTR4	-0.55	5.33E-06	1.28E-04
LTR	ERV1	LTR43-int	-0.6	5.86E-13	1.01E-10
LTR	ERV1	LTR44	-0.5	8.21E-07	2.82E-05
LTR	ERV1	LTR78	-0.63	1.69E-12	2.49E-10
LTR	ERV1	MER52-int	-0.56	3.24E-07	1.52E-05
LTR	ERV1	MER57D	-0.53	1.38E-07	7.92E-06
LTR	ERV1	MER57E1	-0.51	3.25E-08	2.58E-06
LTR	ERV1	MER92C	-0.62	9.55E-08	6.43E-06
LTR	ERV1	PrimLTR79	-0.53	1.19E-07	7.22E-06
LTR	ERVK	LTR5	-0.56	7.80E-05	1.34E-03
LTR	ERVL	HERVL74-int	-0.55	5.45E-07	2.08E-05
LTR	ERVL	LTR40c	-0.62	9.54E-29	9.84E-26
LTR	ERVL	LTR84a	-0.51	2.29E-07	1.24E-05
LTR	ERVL-MaLR	MLT1E1A-int	0.99	1.34E-06	4.19E-05
LTR	ERVL-MaLR	THE1A	-0.76	9.98E-08	6.43E-06
LTR	ERVL-MaLR	THE1C-int	-0.55	3.13E-09	3.23E-07
LTR	Gypsy	LTR81	-0.54	4.24E-07	1.74E-05
RC	Helitron	Helitron1Nb_Mam	-1.19	5.83E-08	4.29E-06
Satellite	centr	GSAT	-0.82	6.43E-05	1.12E-03
Satellite	Satellite	HSAT5	-0.77	2.89E-07	1.42E-05
SINE	Alu	AluSc8	-0.73	3.14E-12	4.04E-10
SINE	Alu	AluSp	-0.53	3.45E-25	1.78E-22

Table S3: Results of the LP vs C comparison with edgeR. List of all elements differentially expressed at FDR ≤ 0.01.

Class	Family	Subfamily	log_2FC	p-value	FDR
DNA	DNA	Eulor1	-0.61	9.47E-04	3.07E-03
DNA	DNA	MER123	-0.44	2.85E-06	2.71E-05
DNA	DNA	MER135	0.82	1.86E-05	1.33E-04
DNA	hAT	MamRep1894	1.24	1.02E-08	2.51E-07
DNA	hAT-Blackjack	MER63C	-1.07	1.68E-04	7.47E-04
DNA	hAT-Blackjack	MER63D	-0.73	2.51E-03	7.04E-03
DNA	hAT-Blackjack	MER94B	-0.55	4.95E-06	4.30E-05
DNA	hAT-Charlie	Chap1_Mam	-0.55	5.42E-05	3.08E-04
DNA	hAT-Charlie	Charlie1	-0.43	3.32E-04	1.31E-03
DNA	hAT-Charlie	Charlie10a	-0.41	3.38E-04	1.33E-03
DNA	hAT-Charlie	Charlie10b	-0.4	1.02E-05	8.30E-05
DNA	hAT-Charlie	Charlie11	-0.39	2.42E-03	6.80E-03
DNA	hAT-Charlie	Charlie12	-0.36	2.29E-04	9.78E-04
DNA	hAT-Charlie	Charlie14a	-0.32	3.46E-03	8.86E-03
DNA	hAT-Charlie	Charlie15b	-0.31	3.39E-03	8.70E-03
DNA	hAT-Charlie	Charlie17	-0.3	8.02E-04	2.66E-03
DNA	hAT-Charlie	Charlie1a	-0.54	9.03E-05	4.70E-04
DNA	hAT-Charlie	Charlie1b	-1.17	1.12E-05	8.86E-05
DNA	hAT-Charlie	Charlie20a	-1.12	2.95E-03	7.74E-03
DNA	hAT-Charlie	Charlie24	-0.66	2.09E-03	5.96E-03
DNA	hAT-Charlie	Charlie25	-0.52	2.57E-05	1.74E-04
DNA	hAT-Charlie	Charlie2b	-0.51	3.15E-03	8.21E-03
DNA	hAT-Charlie	Charlie5	-0.47	2.67E-03	7.32E-03
DNA	hAT-Charlie	Charlie7	-0.47	3.08E-03	8.05E-03
DNA	hAT-Charlie	Charlie7a	-0.42	8.63E-04	2.83E-03
DNA	hAT-Charlie	MER103C	-0.39	2.69E-19	4.63E-17
DNA	hAT-Charlie	MER106B	0.61	1.49E-19	3.08E-17
DNA	hAT-Charlie	MER58C	-0.61	3.06E-03	8.02E-03
DNA	hAT-Charlie	MER58D	-1.25	1.80E-03	5.26E-03
DNA	hAT-Charlie	MER5C	-1.2	2.72E-03	7.38E-03
DNA	hAT-Tip100	Arthur1	-0.96	1.07E-04	5.38E-04
DNA	hAT-Tip100	Arthur1B	-0.92	3.78E-05	2.31E-04
DNA	hAT-Tip100	Arthur1C	-0.9	1.96E-08	4.39E-07
DNA	hAT-Tip100	FordPrefect	-0.89	5.75E-07	6.91E-06
DNA	hAT-Tip100	FordPrefect_a	-0.88	8.22E-08	1.36E-06
DNA	hAT-Tip100	MamTip1	-0.85	3.47E-06	3.12E-05
DNA	hAT-Tip100	MamTip2	-0.84	2.13E-04	9.22E-04
DNA	hAT-Tip100	MER45B	-0.81	1.68E-05	1.24E-04
DNA	hAT-Tip100	MER45C	-0.8	3.29E-04	1.30E-03
DNA	hAT-Tip100	MER45R	-0.78	2.58E-05	1.74E-04
DNA	hAT-Tip100	MER96B	-0.77	2.59E-03	7.23E-03
DNA	hAT-Tip100	MER97b	-0.76	2.60E-15	2.99E-13
DNA	hAT-Tip100	ORSL-2a	-0.74	2.80E-04	1.14E-03
DNA	hAT-Tip100	ORSL-2b	-0.72	3.20E-04	1.28E-03
DNA	hAT-Tip100	Zaphod	-0.72	2.94E-03	7.74E-03
DNA	MULE-MuDR	Ricksha_a	-0.71	5.05E-05	2.90E-04

DNA	PiggyBac	Looper	-0.71	1.04E-05	8.41E-05
DNA	PiggyBac	MER75A	-0.69	2.11E-21	7.25E-19
DNA	TcMar-Mariner	HSMAR2	-0.69	4.93E-04	1.86E-03
DNA	TcMar-Mariner	MADE1	-0.68	1.39E-04	6.43E-04
DNA	TcMar-Tc2	Kanga11a	-0.68	1.56E-04	6.97E-04
DNA	TcMar-Tc2	Kanga1a	-0.67	3.09E-05	2.03E-04
DNA	TcMar-Tc2	Kanga1c	-0.67	2.20E-05	1.54E-04
DNA	TcMar-Tc2	Kanga1d	-0.67	9.83E-04	3.15E-03
DNA	TcMar-Tc2	Kanga2_a	-0.66	3.26E-04	1.30E-03
DNA	TcMar-Tigger	MER127	-0.65	1.32E-04	6.27E-04
DNA	TcMar-Tigger	MER2B	-0.65	1.43E-06	1.50E-05
DNA	TcMar-Tigger	MER47A	-0.64	1.25E-04	6.04E-04
DNA	TcMar-Tigger	MER47B	-0.64	2.25E-03	6.35E-03
DNA	TcMar-Tigger	MERX	-0.63	4.11E-07	5.31E-06
DNA	TcMar-Tigger	Tigger10	-0.63	3.62E-03	9.18E-03
DNA	TcMar-Tigger	Tigger17	-0.62	1.39E-03	4.17E-03
DNA	TcMar-Tigger	Tigger17a	-0.62	2.09E-03	5.96E-03
DNA	TcMar-Tigger	Tigger1a_Mars	-0.62	1.26E-04	6.10E-04
DNA	TcMar-Tigger	Tigger2b_Pri	-0.61	9.99E-06	8.19E-05
DNA	TcMar-Tigger	Tigger3	-0.61	3.34E-05	2.14E-04
DNA	TcMar-Tigger	Tigger3b	-0.6	7.16E-04	2.46E-03
DNA	TcMar-Tigger	Tigger4a	-0.6	2.90E-03	7.69E-03
DNA	TcMar-Tigger	Tigger5	-0.6	3.45E-06	3.12E-05
DNA	TcMar-Tigger	Tigger6a	-0.59	3.90E-06	3.44E-05
DNA	TcMar?	Eulor8	-0.59	2.68E-03	7.32E-03
DNA	TcMar?	MER121	-0.59	2.11E-08	4.55E-07
LINE	CR1	CR1_Mam	-0.59	5.65E-05	3.17E-04
LINE	CR1	L3b	-0.58	2.68E-03	7.32E-03
LINE	CR1	X6A_LINE	-0.58	2.40E-04	1.01E-03
LINE	CR1	X8_LINE	-0.58	3.62E-03	9.18E-03
LINE	Dong-R4	Mam_R4	-0.57	2.36E-11	9.74E-10
LINE	L1	HAL1b	-0.57	2.04E-03	5.87E-03
LINE	L1	HAL1M8	-0.55	4.06E-05	2.47E-04
LINE	L1	HAL1ME	-0.55	3.40E-03	8.72E-03
LINE	L1	L1HS	-0.54	8.31E-08	1.36E-06
LINE	L1	L1M1	-0.54	2.88E-03	7.67E-03
LINE	L1	L1M2a1	-0.54	3.37E-07	4.58E-06
LINE	L1	L1M3	-0.54	7.99E-04	2.66E-03
LINE	L1	L1M3a	-0.54	8.45E-12	3.97E-10
LINE	L1	L1M3b	-0.54	8.68E-05	4.57E-04
LINE	L1	L1M3de	-0.54	1.90E-03	5.48E-03
LINE	L1	L1M3e	-0.53	9.87E-04	3.15E-03
LINE	L1	L1M3f	-0.53	1.81E-12	1.10E-10
LINE	L1	L1M4a2	-0.53	6.82E-04	2.40E-03
LINE	L1	L1M4b	-0.52	4.18E-04	1.61E-03
LINE	L1	L1M4c	-0.52	1.68E-07	2.64E-06
LINE	L1	L1M6	-0.52	3.78E-03	9.48E-03
LINE	L1	L1M7	-0.52	1.09E-04	5.43E-04
LINE	L1	L1MA1	-0.51	9.74E-05	5.02E-04
LINE	L1	L1MA10	-0.51	6.08E-05	3.32E-04

LINE	L1	L1MA3	-0.51	1.36E-04	6.38E-04
LINE	L1	L1MA4	-0.51	2.52E-03	7.05E-03
LINE	L1	L1MA4A	-0.5	1.08E-03	3.38E-03
LINE	L1	L1MA5	-0.5	6.76E-04	2.38E-03
LINE	L1	L1MA5A	-0.49	3.75E-05	2.30E-04
LINE	L1	L1MA6	-0.49	1.05E-04	5.31E-04
LINE	L1	L1MA9	-0.49	7.97E-04	2.66E-03
LINE	L1	L1MB1	-0.49	3.53E-07	4.68E-06
LINE	L1	L1MB2	-0.49	1.53E-08	3.50E-07
LINE	L1	L1MB3	-0.48	1.26E-03	3.87E-03
LINE	L1	L1MB4	-0.47	1.47E-03	4.38E-03
LINE	L1	L1MB5	-0.47	3.27E-03	8.42E-03
LINE	L1	L1MC	-0.47	4.44E-06	3.89E-05
LINE	L1	L1MC1	-0.47	2.84E-03	7.57E-03
LINE	L1	L1MC2	-0.47	9.33E-04	3.04E-03
LINE	L1	L1MCb	-0.47	2.30E-05	1.59E-04
LINE	L1	L1MCc	-0.46	7.57E-04	2.57E-03
LINE	L1	L1MD	-0.46	2.81E-04	1.14E-03
LINE	L1	L1MD1	-0.46	4.23E-04	1.63E-03
LINE	L1	L1MD2	-0.46	1.33E-10	4.91E-09
LINE	L1	L1MD3	-0.46	1.44E-03	4.33E-03
LINE	L1	L1MDa	-0.46	3.07E-03	8.03E-03
LINE	L1	L1MDb	-0.46	2.16E-04	9.29E-04
LINE	L1	L1ME1	-0.46	1.34E-04	6.32E-04
LINE	L1	L1ME2	-0.45	1.90E-03	5.48E-03
LINE	L1	L1ME3A	-0.45	2.78E-03	7.51E-03
LINE	L1	L1ME3B	-0.45	1.47E-04	6.65E-04
LINE	L1	L1ME3C	-0.45	1.75E-04	7.71E-04
LINE	L1	L1ME3E	-0.45	3.63E-03	9.18E-03
LINE	L1	L1MEb	-0.45	3.25E-03	8.41E-03
LINE	L1	L1MEc	-0.45	5.70E-04	2.08E-03
LINE	L1	L1MEd	-0.44	6.99E-04	2.42E-03
LINE	L1	L1MEg1	-0.44	1.85E-07	2.82E-06
LINE	L1	L1MEg2	-0.44	8.45E-05	4.47E-04
LINE	L1	L1MEh	-0.44	1.01E-03	3.21E-03
LINE	L1	L1MEi	-0.44	1.09E-04	5.43E-04
LINE	L1	L1MEj	-0.44	3.66E-05	2.29E-04
LINE	L1	L1P	-0.44	1.42E-06	1.50E-05
LINE	L1	L1P1	-0.43	2.49E-07	3.52E-06
LINE	L1	L1P2	-0.43	2.11E-07	3.03E-06
LINE	L1	L1P3	-0.43	2.88E-04	1.16E-03
LINE	L1	L1P3b	-0.43	1.14E-13	7.84E-12
LINE	L1	L1P4	-0.43	3.33E-06	3.08E-05
LINE	L1	L1P4a	-0.43	5.72E-04	2.08E-03
LINE	L1	L1P4b	-0.43	4.36E-07	5.49E-06
LINE	L1	L1P4c	-0.42	2.72E-03	7.38E-03
LINE	L1	L1P4e	-0.42	4.47E-04	1.71E-03
LINE	L1	L1P5	-0.42	2.49E-05	1.72E-04
LINE	L1	L1PA10	-0.42	7.07E-04	2.44E-03
LINE	L1	L1PA11	-0.42	3.52E-05	2.23E-04

LINE	L1	L1PA12	-0.42	8.44E-06	7.04E-05
LINE	L1	L1PA14	-0.41	4.06E-07	5.30E-06
LINE	L1	L1PA15	-0.41	1.10E-03	3.43E-03
LINE	L1	L1PA15-16	-0.41	1.48E-03	4.39E-03
LINE	L1	L1PA17	-0.41	5.99E-09	1.65E-07
LINE	L1	L1PA2	-0.41	2.71E-06	2.65E-05
LINE	L1	L1PA3	-0.41	9.98E-07	1.12E-05
LINE	L1	L1PA4	-0.41	4.81E-07	5.87E-06
LINE	L1	L1PA5	-0.4	2.60E-05	1.75E-04
LINE	L1	L1PA6	-0.4	3.74E-05	2.30E-04
LINE	L1	L1PA7	-0.4	2.37E-04	1.00E-03
LINE	L1	L1PA8	-0.4	7.75E-05	4.15E-04
LINE	L1	L1PA8A	-0.4	8.72E-05	4.57E-04
LINE	L1	L1PB	-0.4	5.85E-04	2.11E-03
LINE	L1	L1PB1	-0.39	7.58E-04	2.57E-03
LINE	L1	L1PB2	-0.39	2.86E-04	1.16E-03
LINE	L1	L1PBa	-0.39	6.03E-04	2.16E-03
LINE	L1	L1PBa1	-0.39	5.91E-04	2.13E-03
LINE	L1	L1PBb	-0.39	1.91E-07	2.85E-06
LINE	RTE-X	L4_A_Mam	-0.38	3.72E-03	9.38E-03
LINE	RTE-X	L4_C_Mam	-0.38	9.77E-05	5.02E-04
LTR	ERV1	HERV-Fc1_LTR1	-0.38	5.22E-05	2.98E-04
LTR	ERV1	HERV-Fc1-int	-0.38	3.68E-19	5.42E-17
LTR	ERV1	HERV15-int	-0.38	3.70E-06	3.29E-05
LTR	ERV1	HERV3-int	-0.38	1.27E-08	3.06E-07
LTR	ERV1	HERVE_a-int	-0.38	9.43E-04	3.06E-03
LTR	ERV1	HERVE-int	-0.38	1.77E-05	1.29E-04
LTR	ERV1	HERVH-int	-0.38	1.36E-03	4.09E-03
LTR	ERV1	HERVI-int	-0.38	2.30E-04	9.78E-04
LTR	ERV1	HERVP71A-int	-0.37	1.76E-04	7.72E-04
LTR	ERV1	HERVS71-int	-0.37	2.53E-07	3.53E-06
LTR	ERV1	HUERS-P2-int	-0.37	3.41E-08	7.04E-07
LTR	ERV1	HUERS-P3-int	-0.37	7.90E-08	1.36E-06
LTR	ERV1	HUERS-P3b-int	-0.37	1.78E-03	5.21E-03
LTR	ERV1	LOR1-int	-0.37	4.74E-05	2.76E-04
LTR	ERV1	LOR1a	-0.37	1.52E-03	4.51E-03
LTR	ERV1	LOR1b	-0.37	3.50E-03	8.92E-03
LTR	ERV1	LTR109A2	-0.37	2.05E-03	5.90E-03
LTR	ERV1	LTR10B	-0.36	1.11E-06	1.20E-05
LTR	ERV1	LTR10B2	-0.36	6.99E-07	8.20E-06
LTR	ERV1	LTR12	-0.36	4.28E-05	2.55E-04
LTR	ERV1	LTR12_	-0.36	4.26E-08	8.31E-07
LTR	ERV1	LTR12B	-0.36	2.77E-10	9.85E-09
LTR	ERV1	LTR12C	-0.36	2.11E-03	5.99E-03
LTR	ERV1	LTR12D	-0.36	6.44E-05	3.50E-04
LTR	ERV1	LTR12E	-0.36	5.27E-08	9.71E-07
LTR	ERV1	LTR19B	-0.36	1.30E-04	6.19E-04
LTR	ERV1	LTR19C	-0.35	4.12E-05	2.49E-04
LTR	ERV1	LTR1A1	-0.35	1.19E-04	5.81E-04
LTR	ERV1	LTR1B1	-0.35	2.02E-06	2.04E-05

LTR	ERV1	LTR23	-0.35	2.18E-03	6.18E-03
LTR	ERV1	LTR24	-0.35	1.31E-04	6.24E-04
LTR	ERV1	LTR24C	-0.35	6.92E-04	2.41E-03
LTR	ERV1	LTR26E	-0.34	9.61E-04	3.10E-03
LTR	ERV1	LTR27	-0.34	1.32E-03	4.00E-03
LTR	ERV1	LTR34	-0.34	1.82E-05	1.31E-04
LTR	ERV1	LTR36	-0.34	3.84E-04	1.49E-03
LTR	ERV1	LTR37-int	-0.34	2.80E-03	7.54E-03
LTR	ERV1	LTR37A	-0.34	7.20E-06	6.09E-05
LTR	ERV1	LTR37B	-0.34	2.90E-03	7.69E-03
LTR	ERV1	LTR38	-0.34	5.97E-09	1.65E-07
LTR	ERV1	LTR38A1	-0.34	1.02E-03	3.22E-03
LTR	ERV1	LTR38C	-0.34	3.90E-08	7.89E-07
LTR	ERV1	LTR4	-0.33	8.18E-08	1.36E-06
LTR	ERV1	LTR43-int	-0.33	8.94E-14	6.60E-12
LTR	ERV1	LTR44	-0.33	1.98E-06	2.02E-05
LTR	ERV1	LTR45	-0.33	1.66E-03	4.91E-03
LTR	ERV1	LTR45C	-0.32	2.86E-06	2.71E-05
LTR	ERV1	LTR46-int	-0.32	7.28E-09	1.83E-07
LTR	ERV1	LTR49-int	-0.32	4.56E-04	1.74E-03
LTR	ERV1	LTR51	-0.32	1.28E-05	9.96E-05
LTR	ERV1	LTR54B	-0.32	1.09E-03	3.40E-03
LTR	ERV1	LTR64	-0.32	1.07E-04	5.38E-04
LTR	ERV1	LTR68	-0.32	6.01E-05	3.32E-04
LTR	ERV1	LTR6B	-0.31	1.27E-03	3.87E-03
LTR	ERV1	LTR70	-0.31	2.25E-11	9.69E-10
LTR	ERV1	LTR71B	-0.31	2.02E-07	2.98E-06
LTR	ERV1	LTR72B	-0.31	1.10E-05	8.80E-05
LTR	ERV1	LTR76	-0.31	2.93E-05	1.95E-04
LTR	ERV1	LTR77	-0.31	6.35E-08	1.13E-06
LTR	ERV1	LTR78	-0.31	6.80E-21	1.76E-18
LTR	ERV1	LTR7B	-0.31	2.94E-03	7.74E-03
LTR	ERV1	LTR7Y	-0.3	6.33E-04	2.26E-03
LTR	ERV1	LTR8	-0.3	8.36E-04	2.76E-03
LTR	ERV1	LTR8A	-0.3	7.13E-04	2.45E-03
LTR	ERV1	LTR8B	-0.3	1.32E-03	4.00E-03
LTR	ERV1	LTR9A1	-0.3	1.04E-06	1.15E-05
LTR	ERV1	LTR9B	-0.29	5.66E-04	2.08E-03
LTR	ERV1	LTR9D	-0.29	1.53E-04	6.86E-04
LTR	ERV1	MER101-int	-0.29	4.83E-07	5.87E-06
LTR	ERV1	MER110	-0.29	1.97E-14	1.85E-12
LTR	ERV1	MER110A	-0.29	1.30E-06	1.38E-05
LTR	ERV1	MER31B	-0.29	6.44E-06	5.54E-05
LTR	ERV1	MER34	-0.29	1.50E-04	6.78E-04
LTR	ERV1	MER34A	-0.28	2.63E-03	7.30E-03
LTR	ERV1	MER34C_	-0.28	4.56E-08	8.57E-07
LTR	ERV1	MER39	0.34	3.23E-03	8.35E-03
LTR	ERV1	MER39B	0.34	3.48E-05	2.22E-04
LTR	ERV1	MER41-int	0.36	3.18E-04	1.28E-03
LTR	ERV1	MER41B	0.76	3.65E-03	9.22E-03

LTR	ERV1	MER41C	0.85	2.65E-03	7.32E-03
LTR	ERV1	MER41E	-0.83	1.59E-06	1.65E-05
LTR	ERV1	MER49	-0.71	6.76E-05	3.66E-04
LTR	ERV1	MER4A	-0.69	1.42E-07	2.25E-06
LTR	ERV1	MER4B	-0.69	7.08E-05	3.81E-04
LTR	ERV1	MER4B-int	-0.66	6.45E-08	1.13E-06
LTR	ERV1	MER4CL34	-0.65	5.96E-05	3.31E-04
LTR	ERV1	MER4D	-0.64	1.22E-04	5.94E-04
LTR	ERV1	MER4D1	-0.62	3.41E-04	1.33E-03
LTR	ERV1	MER4E	-0.57	3.39E-04	1.33E-03
LTR	ERV1	MER50	-0.56	2.05E-04	8.89E-04
LTR	ERV1	MER50B	-0.55	1.33E-08	3.12E-07
LTR	ERV1	MER51C	-0.52	1.81E-14	1.85E-12
LTR	ERV1	MER52-int	-0.52	3.97E-09	1.21E-07
LTR	ERV1	MER52C	-0.52	3.13E-09	9.79E-08
LTR	ERV1	MER57-int	-0.51	1.38E-03	4.17E-03
LTR	ERV1	MER57A-int	-0.51	2.64E-03	7.31E-03
LTR	ERV1	MER57A1	-0.5	6.73E-07	7.99E-06
LTR	ERV1	MER57C2	-0.49	1.12E-04	5.55E-04
LTR	ERV1	MER57D	-0.49	2.64E-12	1.36E-10
LTR	ERV1	MER57E1	-0.49	1.19E-10	4.60E-09
LTR	ERV1	MER57E2	-0.48	3.12E-06	2.93E-05
LTR	ERV1	MER57E3	-0.47	3.15E-07	4.33E-06
LTR	ERV1	MER61C	-0.45	8.61E-04	2.83E-03
LTR	ERV1	MER61D	-0.45	2.82E-03	7.57E-03
LTR	ERV1	MER61E	-0.44	5.28E-04	1.96E-03
LTR	ERV1	MER65-int	-0.43	8.08E-06	6.79E-05
LTR	ERV1	MER66A	-0.43	2.83E-03	7.57E-03
LTR	ERV1	MER66C	-0.41	7.72E-04	2.61E-03
LTR	ERV1	MER67A	-0.41	9.16E-07	1.04E-05
LTR	ERV1	MER67C	-0.4	5.80E-05	3.24E-04
LTR	ERV1	MER67D	-0.4	1.23E-03	3.79E-03
LTR	ERV1	MER84-int	-0.4	7.89E-07	9.06E-06
LTR	ERV1	MER92C	-0.4	6.59E-09	1.74E-07
LTR	ERV1	MER95	-0.4	8.54E-06	7.05E-05
LTR	ERV1	PABL_A-int	-0.4	9.63E-04	3.10E-03
LTR	ERV1	PABL_B	-0.39	2.56E-05	1.74E-04
LTR	ERV1	PRIMA4_LTR	-0.39	6.74E-04	2.38E-03
LTR	ERV1	PRIMA4-int	-0.39	1.35E-05	1.01E-04
LTR	ERV1	PRIMA41-int	-0.38	1.36E-05	1.01E-04
LTR	ERV1	PRIMAX-int	-0.38	8.71E-04	2.85E-03
LTR	ERV1	PrimLTR79	-0.38	3.07E-14	2.65E-12
LTR	ERVK	HERVK-int	-0.37	1.44E-11	6.47E-10
LTR	ERVK	HERVK11-int	-0.37	2.06E-15	2.66E-13
LTR	ERVK	HERVK13-int	-0.37	3.26E-05	2.10E-04
LTR	ERVK	HERVK14-int	-0.37	9.96E-05	5.09E-04
LTR	ERVK	HERVK3-int	-0.37	3.26E-05	2.10E-04
LTR	ERVK	HERVK9-int	-0.37	1.43E-04	6.55E-04
LTR	ERVK	HERVKC4-int	-0.37	1.13E-04	5.58E-04
LTR	ERVK	LTR22B2	-0.37	6.76E-09	1.74E-07

LTR	ERVK	LTR22C	-0.36	1.75E-03	5.15E-03
LTR	ERVK	LTR22C2	-0.36	4.94E-04	1.86E-03
LTR	ERVK	LTR3	-0.36	2.67E-03	7.32E-03
LTR	ERVK	LTR5	-0.36	4.73E-05	2.76E-04
LTR	ERVK	LTR5A	-0.35	6.67E-06	5.70E-05
LTR	ERVK	MER11B	-0.35	3.91E-03	9.75E-03
LTR	ERVK	MER11C	-0.35	1.12E-03	3.48E-03
LTR	ERVK	MER11D	-0.35	2.61E-03	7.27E-03
LTR	ERVK	MER9B	-0.34	2.65E-04	1.09E-03
LTR	ERVL	ERVL-B4-int	-0.34	3.44E-04	1.34E-03
LTR	ERVL	ERVL-E-int	-0.34	6.08E-05	3.32E-04
LTR	ERVL	ERVL-int	-0.33	2.78E-04	1.14E-03
LTR	ERVL	ERVL47-int	-0.33	3.21E-04	1.28E-03
LTR	ERVL	HERV16-int	-0.33	4.33E-07	5.49E-06
LTR	ERVL	HERVL32-int	-0.33	2.08E-08	4.55E-07
LTR	ERVL	HERVL66-int	-0.33	4.65E-04	1.77E-03
LTR	ERVL	HERVL74-int	-0.33	1.20E-10	4.60E-09
LTR	ERVL	LTR16	-0.33	3.08E-08	6.50E-07
LTR	ERVL	LTR16A1	-0.33	5.42E-04	2.01E-03
LTR	ERVL	LTR16A2	-0.33	2.34E-06	2.35E-05
LTR	ERVL	LTR16B	-0.32	1.75E-07	2.70E-06
LTR	ERVL	LTR16B2	-0.32	2.07E-05	1.46E-04
LTR	ERVL	LTR16D	-0.32	2.58E-04	1.07E-03
LTR	ERVL	LTR16D2	-0.32	1.39E-04	6.43E-04
LTR	ERVL	LTR16E1	-0.32	5.67E-04	2.08E-03
LTR	ERVL	LTR18A	-0.32	3.61E-05	2.28E-04
LTR	ERVL	LTR18C	-0.31	4.23E-05	2.54E-04
LTR	ERVL	LTR33C	-0.31	1.21E-05	9.47E-05
LTR	ERVL	LTR40b	-0.31	7.95E-04	2.66E-03
LTR	ERVL	LTR40c	-0.31	3.51E-37	3.63E-34
LTR	ERVL	LTR41C	-0.31	1.11E-06	1.20E-05
LTR	ERVL	LTR47A	-0.31	3.33E-06	3.08E-05
LTR	ERVL	LTR47A2	-0.3	5.60E-05	3.16E-04
LTR	ERVL	LTR47B2	-0.3	7.73E-10	2.66E-08
LTR	ERVL	LTR53	-0.29	3.37E-06	3.08E-05
LTR	ERVL	LTR57	-0.29	1.85E-03	5.37E-03
LTR	ERVL	LTR57-int	-0.29	7.57E-12	3.72E-10
LTR	ERVL	LTR66	-0.29	1.64E-03	4.86E-03
LTR	ERVL	LTR67B	-0.29	1.16E-03	3.57E-03
LTR	ERVL	LTR75B	-0.29	1.19E-03	3.68E-03
LTR	ERVL	LTR80B	-0.28	1.34E-05	1.01E-04
LTR	ERVL	LTR83	-0.28	2.47E-06	2.46E-05
LTR	ERVL	LTR84a	-0.28	6.06E-09	1.65E-07
LTR	ERVL	MER54A	-0.28	1.91E-04	8.38E-04
LTR	ERVL	MER74C	-0.28	3.24E-05	2.10E-04
LTR	ERVL	MER76-int	-0.28	5.69E-04	2.08E-03
LTR	ERVL	MLT2B1	-1.26	6.89E-04	2.41E-03
LTR	ERVL	MLT2B2	-1.01	1.84E-05	1.32E-04
LTR	ERVL	MLT2B4	-1	5.76E-04	2.09E-03
LTR	ERVL	MLT2F	-0.89	4.97E-05	2.87E-04

LTR	ERVL-MaLR	MLT1A-int	-0.87	8.13E-04	2.69E-03
LTR	ERVL-MaLR	MLT1A0-int	-0.66	2.70E-03	7.37E-03
LTR	ERVL-MaLR	MLT1B	-0.64	2.45E-04	1.02E-03
LTR	ERVL-MaLR	MLT1E	-0.62	5.02E-04	1.88E-03
LTR	ERVL-MaLR	MLT1E1A-int	-0.62	2.07E-07	3.01E-06
LTR	ERVL-MaLR	MLT1E2	-0.6	1.03E-03	3.25E-03
LTR	ERVL-MaLR	MLT1F1-int	-0.59	2.70E-09	8.71E-08
LTR	ERVL-MaLR	MLT1F2-int	-0.58	1.36E-04	6.38E-04
LTR	ERVL-MaLR	MLT1G3-int	-0.56	1.57E-05	1.16E-04
LTR	ERVL-MaLR	MLT1H1-int	-0.54	6.68E-04	2.38E-03
LTR	ERVL-MaLR	MLT1H2-int	-0.51	2.30E-04	9.78E-04
LTR	ERVL-MaLR	MLT1I	-0.5	2.73E-03	7.39E-03
LTR	ERVL-MaLR	MLT1J-int	-0.49	1.76E-03	5.15E-03
LTR	ERVL-MaLR	MLT1J2	-0.49	4.76E-04	1.80E-03
LTR	ERVL-MaLR	MLT1J2-int	-0.48	1.90E-03	5.48E-03
LTR	ERVL-MaLR	MLT1N2	-0.48	2.49E-04	1.04E-03
LTR	ERVL-MaLR	MLT1O	-0.48	1.28E-04	6.13E-04
LTR	ERVL-MaLR	MSTA-int	-0.48	6.70E-04	2.38E-03
LTR	ERVL-MaLR	MSTA1	-0.48	6.89E-04	2.41E-03
LTR	ERVL-MaLR	MSTB-int	-0.47	2.68E-06	2.63E-05
LTR	ERVL-MaLR	MSTB2	-0.46	1.07E-03	3.36E-03
LTR	ERVL-MaLR	MSTC-int	-0.45	1.42E-04	6.53E-04
LTR	ERVL-MaLR	THE1A	-0.44	2.41E-12	1.31E-10
LTR	ERVL-MaLR	THE1B-int	-0.43	7.33E-04	2.51E-03
LTR	ERVL-MaLR	THE1C-int	-0.43	1.44E-04	6.57E-04
LTR	ERVL-MaLR	THE1D	-0.43	3.19E-03	8.28E-03
LTR	ERVL?	LTR89	-0.42	2.42E-04	1.01E-03
LTR	Gypsy	LTR81	-0.42	2.29E-12	1.31E-10
LTR	Gypsy	LTR81A	-0.41	5.85E-08	1.06E-06
LTR	Gypsy	LTR81AB	-0.41	1.72E-05	1.26E-04
LTR	Gypsy	LTR81B	-0.4	1.33E-05	1.01E-04
LTR	Gypsy	LTR81C	-0.4	4.30E-05	2.55E-04
LTR	Gypsy	MamGypLTR1a	-0.4	7.76E-04	2.62E-03
LTR	Gypsy	MamGypLTR1c	-0.39	4.51E-07	5.62E-06
LTR	Gypsy	MamGypLTR2c	-0.39	9.95E-08	1.61E-06
LTR	Gypsy	MamGypLTR3a	-0.39	5.22E-04	1.95E-03
LTR	Gypsy?	LTR85c	-0.39	4.81E-05	2.79E-04
LTR	LTR	LTR106_Mam	-0.38	7.81E-04	2.63E-03
LTR	LTR	LTR107_Mam	-0.38	1.03E-04	5.26E-04
LTR	LTR	MamRep1527	-0.38	5.31E-09	1.57E-07
RC	Helitron	Helitron1Nb_Mam	-0.37	1.15E-09	3.84E-08
Satellite	acro	ACRO1	-0.37	1.94E-04	8.45E-04
Satellite	centr	ALR_Alpha	-0.37	3.84E-03	9.61E-03
Satellite	centr	HSAT4	-0.36	1.34E-05	1.01E-04
Satellite	centr	SST1	-0.36	1.39E-04	6.43E-04
Satellite	Satellite	BSR_Beta	-0.35	9.05E-05	4.70E-04
Satellite	Satellite	CER	-0.35	2.75E-06	2.65E-05
Satellite	Satellite	D20S16	-0.35	2.39E-03	6.74E-03
Satellite	Satellite	MSR1	-0.34	1.08E-06	1.19E-05
Satellite	Satellite	SATR1	-0.34	8.08E-05	4.30E-04

Satellite	Satellite	SATR2	-0.33	2.52E-04	1.04E-03
scRNA	scRNA	HY3	-0.33	4.49E-08	8.57E-07
SINE	Alu	AluSc	-0.32	3.74E-05	2.30E-04
SINE	Alu	AluSc8	-0.32	8.31E-14	6.60E-12
SINE	Alu	AluSg4	-0.32	1.33E-03	4.03E-03
SINE	Alu	AluSp	-0.31	1.37E-27	7.06E-25
SINE	Alu	AluYb8	-0.31	1.92E-05	1.36E-04
SINE	Alu	AluYb9	-0.3	3.74E-03	9.40E-03
SINE	Alu	AluYc3	-0.3	4.03E-08	8.01E-07
SINE	Alu	AluYf4	-0.3	9.74E-04	3.13E-03
SINE	Alu	AluYg6	-0.3	4.73E-05	2.76E-04
SINE	Alu	AluYh9	-0.3	3.51E-07	4.68E-06
SINE	Alu	AluYk11	-0.29	1.73E-04	7.68E-04
SINE	Alu	AluYk12	-0.29	1.01E-03	3.21E-03
snRNA	snRNA	U14	-0.29	3.01E-05	1.99E-04
snRNA	snRNA	U17	-0.29	1.54E-12	9.96E-11
snRNA	snRNA	U6	-0.28	1.12E-05	8.86E-05
snRNA	snRNA	U7	-0.28	7.89E-07	9.06E-06

Table S4: Results of the NLP vs C comparison with edgeR. List of all elements differentially expressed at FDR ≤ 0.01.

Class	Family	Subfamily	log_2FC	p-value	FDR
DNA	hAT	MamRep1894	-0.48	3.11E-06	8.43E-05
DNA	hAT-Charlie	Charlie1b	-0.36	6.40E-05	1.12E-03
DNA	hAT-Charlie	MER103C	-1.01	8.45E-16	1.74E-13
DNA	hAT-Charlie	MER106B	-0.67	1.46E-17	3.77E-15
DNA	hAT-Tip100	Arthur1C	-0.47	3.92E-07	1.74E-05
DNA	hAT-Tip100	FordPrefect	-0.44	3.30E-06	8.73E-05
DNA	hAT-Tip100	FordPrefect_a	-0.34	5.76E-07	2.12E-05
DNA	hAT-Tip100	MER97b	-0.5	1.31E-08	1.23E-06
DNA	PiggyBac	MER75A	0.66	1.24E-18	4.27E-16
DNA	TcMar-Tigger	MER2B	-0.7	4.12E-04	5.73E-03
DNA	TcMar-Tigger	MERX	-0.54	7.74E-04	8.87E-03
DNA	TcMar?	MER121	-1.07	5.53E-05	1.00E-03
LINE	Dong-R4	Mam_R4	-0.78	4.40E-07	1.74E-05
LINE	L1	HAL1M8	-0.67	6.56E-04	7.86E-03
LINE	L1	L1M3a	0.57	9.24E-07	3.07E-05
LINE	L1	L1M3f	-0.55	1.86E-08	1.60E-06
LINE	L1	L1M4c	-0.6	7.58E-06	1.74E-04
LINE	L1	L1MB2	-0.5	2.68E-07	1.38E-05
LINE	L1	L1MD2	-0.63	6.04E-07	2.15E-05
LINE	L1	L1MEg1	-0.56	2.61E-04	3.90E-03
LINE	L1	L1P3b	-0.53	1.93E-11	2.21E-09
LINE	L1	L1P5	-0.51	1.71E-04	2.72E-03
LINE	L1	L1PA14	-0.62	3.41E-06	8.78E-05
LINE	L1	L1PA17	-0.53	1.41E-06	4.26E-05
LINE	L1	L1PBb	-0.56	1.06E-06	3.42E-05
LTR	ERV1	ERV24B_Prim-int	-0.55	2.43E-05	4.82E-04
LTR	ERV1	HERV3-int	-0.46	6.26E-04	7.68E-03
LTR	ERV1	LOR1-int	-0.48	2.88E-04	4.18E-03
LTR	ERV1	LTR10B	-0.44	1.52E-06	4.47E-05
LTR	ERV1	LTR10B2	-0.49	5.66E-04	7.21E-03
LTR	ERV1	LTR12B	-0.62	2.71E-04	4.00E-03
LTR	ERV1	LTR19B	-0.47	5.10E-04	6.66E-03
LTR	ERV1	LTR1B1	-0.46	6.68E-06	1.57E-04
LTR	ERV1	LTR38	-0.42	1.76E-05	3.64E-04
LTR	ERV1	LTR38C	-0.46	2.19E-04	3.36E-03
LTR	ERV1	LTR4	-0.41	5.33E-06	1.28E-04
LTR	ERV1	LTR43-int	-0.51	5.86E-13	1.01E-10
LTR	ERV1	LTR44	-0.48	8.21E-07	2.82E-05
LTR	ERV1	LTR45C	0.45	3.10E-05	5.91E-04
LTR	ERV1	LTR51	-0.44	1.95E-04	3.04E-03
LTR	ERV1	LTR64	-0.4	4.52E-04	6.05E-03
LTR	ERV1	LTR68	-0.41	5.90E-04	7.33E-03
LTR	ERV1	LTR78	0.99	1.69E-12	2.49E-10
LTR	ERV1	MER101-int	-0.44	3.61E-04	5.10E-03
LTR	ERV1	MER110	-0.36	1.87E-05	3.78E-04
LTR	ERV1	MER110A	-0.44	4.55E-06	1.14E-04

LTR	ERV1	MER34C_	-0.39	1.21E-05	2.59E-04
LTR	ERV1	MER4A	-0.45	3.60E-05	6.76E-04
LTR	ERV1	MER4B-int	-0.42	9.87E-06	2.17E-04
LTR	ERV1	MER4CL34	-0.34	7.93E-04	8.99E-03
LTR	ERV1	MER52-int	-0.37	3.24E-07	1.52E-05
LTR	ERV1	MER52C	-0.34	1.14E-04	1.92E-03
LTR	ERV1	MER57A1	-0.36	5.66E-04	7.21E-03
LTR	ERV1	MER57C2	-0.37	7.71E-04	8.87E-03
LTR	ERV1	MER57D	-0.33	1.38E-07	7.92E-06
LTR	ERV1	MER57E1	-0.37	3.25E-08	2.58E-06
LTR	ERV1	MER65-int	0.4	1.46E-04	2.39E-03
LTR	ERV1	MER65B	-0.34	4.67E-06	1.15E-04
LTR	ERV1	MER67A	-0.33	4.42E-04	6.00E-03
LTR	ERV1	MER84	-0.35	7.14E-04	8.36E-03
LTR	ERV1	MER92C	-0.43	9.55E-08	6.43E-06
LTR	ERV1	PrimLTR79	-0.32	1.19E-07	7.22E-06
LTR	ERVK	LTR5	-0.33	7.80E-05	1.34E-03
LTR	ERVK	MER11D	-0.36	4.19E-04	5.76E-03
LTR	ERVL	HERV16-int	-0.36	1.51E-05	3.17E-04
LTR	ERVL	HERVL74-int	-0.34	5.45E-07	2.08E-05
LTR	ERVL	LTR16	-0.76	5.85E-04	7.33E-03
LTR	ERVL	LTR16D	-0.55	6.49E-04	7.86E-03
LTR	ERVL	LTR40c	-0.5	9.54E-29	9.84E-26
LTR	ERVL	LTR57-int	-0.54	1.54E-04	2.48E-03
LTR	ERVL	LTR84a	-0.48	2.29E-07	1.24E-05
LTR	ERVL-MaLR	MLT1E1A-int	-0.47	1.34E-06	4.19E-05
LTR	ERVL-MaLR	MLT1F1-int	-0.47	2.48E-05	4.82E-04
LTR	ERVL-MaLR	MLT1G3-int	-0.46	3.46E-04	4.96E-03
LTR	ERVL-MaLR	THE1A	-0.45	9.98E-08	6.43E-06
LTR	ERVL-MaLR	THE1C-int	-0.46	3.13E-09	3.23E-07
LTR	ERVL?	LTR89	-0.39	6.90E-04	8.17E-03
LTR	Gypsy	LTR81	-0.36	4.24E-07	1.74E-05
LTR	Gypsy	MamGypLTR2c	-0.32	2.03E-06	5.66E-05
LTR	LTR	MamRep1527	-1.19	8.43E-06	1.89E-04
RC	Helitron	Helitron1Nb_Mam	-0.82	5.83E-08	4.29E-06
Satellite	centr	GSAT	-0.77	6.43E-05	1.12E-03
Satellite	Satellite	HSAT5	-0.73	2.89E-07	1.42E-05
scRNA	scRNA	HY3	-0.53	4.39E-07	1.74E-05
SINE	Alu	AluSc	-0.5	4.66E-04	6.16E-03
SINE	Alu	AluSc8	-0.49	3.14E-12	4.04E-10
SINE	Alu	AluSp	-0.45	3.45E-25	1.78E-22
SINE	Alu	AluYc3	-0.42	2.01E-06	5.66E-05
SINE	Alu	AluYh9	-0.38	4.31E-05	7.94E-04
snRNA	snRNA	U6	-0.35	2.37E-04	3.59E-03
snRNA	snRNA	U7	-0.38	1.39E-04	2.31E-03

Table S5: Results of the family-level LP vs C comparison with edgeR. List of all families differentially expressed at FDR ≤ 0.01.

Class	Family	log_2FC	p-value	FDR
DNA	DNA	-0.32	3.26E-03	9.16E-03
DNA	hAT-Tip100	-0.29	2.37E-03	7.12E-03
DNA	PiggyBac	-0.35	4.55E-04	2.05E-03
DNA	TcMar-Mariner	-0.3	1.36E-03	5.11E-03
DNA	TcMar-Tc2	-0.33	5.90E-04	2.41E-03
DNA	TcMar?	-0.59	8.86E-08	1.99E-06
LINE	Dong-R4	-0.71	1.84E-10	8.28E-09
LINE	RTE-X	-0.28	3.66E-03	9.70E-03
LTR	ERVL?	-0.3	2.34E-03	7.12E-03
LTR	Gypsy	-0.39	5.20E-05	4.68E-04
LTR	LTR	-0.41	6.51E-05	4.89E-04
RC	Helitron	-0.46	4.53E-06	5.10E-05
Satellite	acro	-1.12	2.13E-04	1.06E-03
Satellite	centr	-0.6	1.19E-04	6.84E-04
Satellite	Satellite	-1.02	1.22E-04	6.84E-04
scRNA	scRNA	-0.46	4.13E-06	5.10E-05
snRNA	snRNA	-0.32	1.56E-03	5.38E-03

Table S6: Results of the family-level NLP vs C comparison with edgeR. List of all families differentially expressed at FDR ≤ 0.01.

Class	Family	log_2FC	p-value	FDR
DNA	TcMar?	-0.41	7.61E-05	1.40E-03
LINE	Dong-R4	-0.53	5.91E-07	2.66E-05
scRNA	scRNA	-0.38	9.36E-05	1.40E-03

Table S7: Results of the pairwise comparison with edgeR. List of all elements differentially expressed at FDR ≤ 0.01 with $log_2FC \leq -0.5$ or $log_2FC \geq 0.5$ (translating into foldchange difference greater than 0.71 or 1.41).

Class	Family	Subfamily	log_2FC	p-value	FDR
DNA	DNA	Eulor1	-0.86	9.89E-07	7.91E-06
DNA	hAT-Charlie	Charlie12	-0.72	2.85E-04	7.50E-04
DNA	MULE-MuDR	Ricksha_a	-0.55	4.48E-05	1.71E-04
DNA	TcMar-Tigger	MER127	-0.55	3.42E-09	1.12E-07
DNA	TcMar-Tigger	Tigger1a_Mars	-0.73	1.39E-06	1.04E-05
LINE	L1	L1M2a1	-0.57	1.12E-10	8.29E-09
LINE	L1	L1P4c	-0.6	2.42E-03	4.56E-03
LTR	ERV1	HERV-Fc1_LTR1	-1.02	5.14E-06	2.89E-05
LTR	ERV1	HERV-Fc1-int	-1.17	2.06E-14	5.31E-12
LTR	ERV1	HERVI-int	-0.62	3.06E-07	3.33E-06
LTR	ERV1	HERVS71-int	-0.61	8.23E-10	4.04E-08
LTR	ERV1	LTR06	-0.59	7.57E-07	6.51E-06
LTR	ERV1	LTR10B2	-0.97	6.83E-06	3.71E-05
LTR	ERV1	LTR26E	-0.51	2.91E-07	3.26E-06
LTR	ERV1	LTR38A1	-0.5	5.02E-06	2.83E-05
LTR	ERV1	LTR46	-0.5	2.47E-09	8.94E-08
LTR	ERV1	LTR46-int	-0.63	3.87E-11	3.52E-09
LTR	ERV1	LTR6B	-0.54	1.05E-08	2.57E-07
LTR	ERV1	LTR70	-0.5	2.30E-11	2.37E-09
LTR	ERV1	LTR71B	-0.73	6.04E-14	1.25E-11
LTR	ERV1	LTR77	-0.87	2.16E-11	2.37E-09
LTR	ERV1	MER51C	0.64	1.65E-05	7.38E-05
LTR	ERV1	MER57E2	-0.7	6.90E-07	6.19E-06
LTR	ERV1	MER61E	-0.5	1.24E-07	1.54E-06
LTR	ERV1	MER65B	0.61	1.06E-07	1.38E-06
LTR	ERV1	MER84-int	-0.55	4.42E-09	1.23E-07
LTR	ERV1	MER95	-0.53	2.67E-06	1.73E-05
LTR	ERVK	HERVK-int	-0.68	1.42E-15	4.93E-13
LTR	ERVK	HERVK11-int	-0.92	4.62E-11	3.67E-09
LTR	ERVK	LTR22	-0.5	6.70E-05	2.30E-04
LTR	ERVK	LTR22B2	-0.95	4.29E-08	7.15E-07
LTR	ERVK	LTR22C	-0.79	6.21E-05	2.18E-04
LTR	ERVK	LTR22C2	-0.67	4.32E-09	1.23E-07
LTR	ERVK	LTR5A	-0.62	1.13E-15	4.93E-13
LTR	ERVL	HERVL32-int	-0.97	1.43E-15	4.93E-13
LTR	ERVL	LTR18C	-0.5	7.17E-04	1.63E-03
LTR	ERVL	LTR47B2	-0.71	1.04E-08	2.57E-07
LTR	ERVL	LTR53	-0.5	2.88E-10	1.86E-08
LTR	ERVL	LTR83	-0.51	4.09E-11	3.52E-09
LTR	ERVL	LTR91	-0.5	1.90E-06	1.33E-05
LTR	ERVL	MER74C	-0.61	3.12E-08	5.55E-07
LTR	ERVL	MER76-int	-0.56	2.49E-09	8.94E-08
LTR	ERVL-MaLR	MLT1H1-int	-0.52	2.21E-09	8.78E-08
LTR	ERVL-MaLR	MSTA1-int	-0.8	1.09E-04	3.46E-04
LTR	Gypsy	LTR81A	-0.51	1.69E-11	2.18E-09

LTR	Gypsy	LTR81AB	-0.53	1.74E-05	7.66E-05
LTR	LTR	LTR107_Mam	-0.51	8.74E-08	1.23E-06
RNA	RNA	7SK	-0.54	3.72E-03	6.52E-03
Satellite	acro	ACRO1	-0.61	1.73E-05	7.66E-05
Satellite	Satellite	_CATTC_n	-0.86	1.09E-04	3.46E-04
Satellite	Satellite	_GAATG_n	-0.74	4.71E-05	1.79E-04
snRNA	snRNA	U17	0.93	2.37E-06	1.55E-05

Table S8: Results of the pairwise comparison with edgeR. List of all elements differentially expressed at FDR ≤ 0.01.

Class	Family	Subfamily	log_2FC	p-value	FDR
DNA	DNA	Eulor1	-0.86	9.89E-07	7.91E-06
DNA	DNA	MER126	-0.34	5.00E-06	2.83E-05
DNA	DNA	MER135	-0.44	6.22E-08	9.45E-07
DNA	hAT	MamRep38	-0.15	2.99E-03	5.41E-03
DNA	hAT	MER53	-0.15	2.33E-03	4.43E-03
DNA	hAT-Blackjack	MER63A	-0.22	2.71E-05	1.13E-04
DNA	hAT-Blackjack	MER63B	-0.29	3.06E-07	3.33E-06
DNA	hAT-Blackjack	MER63C	-0.16	1.29E-03	2.68E-03
DNA	hAT-Blackjack	MER63D	-0.28	3.88E-06	2.29E-05
DNA	hAT-Blackjack	MER81	-0.19	3.00E-04	7.82E-04
DNA	hAT-Blackjack	MER94	-0.2	6.52E-05	2.25E-04
DNA	hAT-Blackjack	MER94B	-0.23	8.85E-04	1.94E-03
DNA	hAT-Charlie	Charlie10b	-0.21	3.46E-03	6.13E-03
DNA	hAT-Charlie	Charlie11	-0.38	2.97E-05	1.22E-04
DNA	hAT-Charlie	Charlie12	-0.72	2.85E-04	7.50E-04
DNA	hAT-Charlie	Charlie13a	-0.26	1.89E-06	1.33E-05
DNA	hAT-Charlie	Charlie14a	-0.3	1.18E-06	9.25E-06
DNA	hAT-Charlie	Charlie15a	-0.13	4.80E-03	8.18E-03
DNA	hAT-Charlie	Charlie15b	-0.16	5.32E-04	1.26E-03
DNA	hAT-Charlie	Charlie16a	-0.17	3.47E-04	8.89E-04
DNA	hAT-Charlie	Charlie17	-0.38	4.01E-09	1.18E-07
DNA	hAT-Charlie	Charlie17a	-0.24	4.12E-06	2.41E-05
DNA	hAT-Charlie	Charlie17b	-0.27	6.01E-05	2.12E-04
DNA	hAT-Charlie	Charlie19a	-0.2	7.09E-05	2.41E-04
DNA	hAT-Charlie	Charlie1a	-0.18	1.82E-04	5.31E-04
DNA	hAT-Charlie	Charlie20a	-0.28	4.56E-07	4.57E-06
DNA	hAT-Charlie	Charlie21a	-0.23	2.85E-05	1.18E-04
DNA	hAT-Charlie	Charlie22a	-0.2	6.29E-05	2.19E-04
DNA	hAT-Charlie	Charlie24	-0.15	1.50E-03	3.03E-03
DNA	hAT-Charlie	Charlie25	-0.18	4.85E-04	1.16E-03
DNA	hAT-Charlie	Charlie2a	-0.19	1.07E-04	3.44E-04
DNA	hAT-Charlie	Charlie2b	-0.15	9.83E-04	2.10E-03
DNA	hAT-Charlie	Charlie3	-0.18	4.06E-04	1.01E-03
DNA	hAT-Charlie	Charlie5	-0.24	2.88E-06	1.82E-05
DNA	hAT-Charlie	Charlie7a	-0.33	6.01E-09	1.59E-07
DNA	hAT-Charlie	Charlie8	-0.24	3.10E-06	1.91E-05
DNA	hAT-Charlie	Charlie9	-0.15	3.44E-03	6.09E-03
DNA	hAT-Charlie	Cheshire	-0.16	8.18E-04	1.82E-03
DNA	hAT-Charlie	MER102b	-0.18	2.89E-04	7.58E-04
DNA	hAT-Charlie	MER105	-0.2	5.05E-05	1.87E-04
DNA	hAT-Charlie	MER106A	-0.29	4.49E-06	2.60E-05
DNA	hAT-Charlie	MER112	-0.14	4.09E-03	7.10E-03
DNA	hAT-Charlie	MER119	-0.18	2.95E-04	7.70E-04
DNA	hAT-Charlie	MER1B	-0.18	3.08E-04	7.98E-04
DNA	hAT-Charlie	MER3	-0.14	3.23E-03	5.77E-03
DNA	hAT-Charlie	MER30B	-0.24	1.08E-03	2.27E-03

DNA	hAT-Charlie	MER58A	-0.17	3.87E-04	9.73E-04
DNA	hAT-Charlie	MER58B	-0.2	5.25E-05	1.92E-04
DNA	hAT-Charlie	MER58D	-0.2	1.88E-04	5.40E-04
DNA	hAT-Charlie	MER5A	-0.16	9.06E-04	1.98E-03
DNA	hAT-Charlie	MER5A1	-0.16	1.03E-03	2.18E-03
DNA	hAT-Charlie	MER5C	-0.26	6.62E-07	6.13E-06
DNA	hAT-Charlie	MER5C1	-0.15	2.98E-03	5.41E-03
DNA	hAT-Tip100	Arthur1	-0.14	4.66E-03	7.98E-03
DNA	hAT-Tip100	Arthur1A	-0.21	2.47E-04	6.73E-04
DNA	hAT-Tip100	Arthur1B	-0.2	2.01E-04	5.62E-04
DNA	hAT-Tip100	MamRep488	-0.33	3.30E-05	1.31E-04
DNA	hAT-Tip100	MamTip1	-0.17	9.72E-04	2.09E-03
DNA	hAT-Tip100	MamTip2	-0.21	1.85E-05	8.10E-05
DNA	hAT-Tip100	MER45A	-0.19	1.25E-04	3.88E-04
DNA	hAT-Tip100	MER45B	-0.19	5.34E-04	1.26E-03
DNA	hAT-Tip100	MER45C	-0.37	1.32E-08	2.96E-07
DNA	hAT-Tip100	MER45R	-0.34	2.38E-06	1.55E-05
DNA	hAT-Tip100	MER91C	-0.27	3.41E-06	2.05E-05
DNA	hAT-Tip100	MER96	-0.24	5.90E-05	2.09E-04
DNA	hAT-Tip100	MER97b	-0.26	3.03E-03	5.46E-03
DNA	hAT-Tip100	MER97d	-0.45	8.10E-07	6.82E-06
DNA	hAT-Tip100	ORSL-2a	-0.24	6.56E-04	1.51E-03
DNA	hAT-Tip100	Zaphod	-0.19	2.70E-04	7.20E-04
DNA	hAT-Tip100	Zaphod3	-0.17	1.41E-03	2.88E-03
DNA	hAT-Tip100?	hAT-N1_Mam	-0.17	5.02E-03	8.48E-03
DNA	hAT-Tip100?	MamRep1879	-0.19	1.67E-03	3.34E-03
DNA	hAT?	MER99	-0.34	7.55E-06	3.96E-05
DNA	Merlin	Merlin1_HS	-0.25	3.52E-03	6.22E-03
DNA	MULE-MuDR	Ricksha	-0.22	1.70E-03	3.38E-03
DNA	MULE-MuDR	Ricksha_0	-0.21	7.27E-04	1.65E-03
DNA	MULE-MuDR	Ricksha_a	-0.55	4.48E-05	1.71E-04
DNA	MULE-MuDR	Ricksha_b	-0.32	1.09E-04	3.46E-04
DNA	MULE-MuDR	Ricksha_c	-0.25	3.11E-06	1.91E-05
DNA	PiggyBac	Looper	-0.23	9.31E-06	4.64E-05
DNA	PiggyBac	MER75	-0.16	3.39E-03	6.02E-03
DNA	PiggyBac	MER85	-0.17	4.44E-03	7.63E-03
DNA	TcMar-Mariner	HSMAR1	-0.19	1.21E-04	3.76E-04
DNA	TcMar-Mariner	HSMAR2	-0.24	1.94E-06	1.33E-05
DNA	TcMar-Mariner	MADE2	-0.23	8.20E-06	4.21E-05
DNA	TcMar-Mariner	MARNA	-0.16	4.05E-04	1.01E-03
DNA	TcMar-Tc2	Kanga11a	-0.25	1.93E-06	1.33E-05
DNA	TcMar-Tc2	Kanga1a	-0.19	2.35E-04	6.45E-04
DNA	TcMar-Tc2	Kanga1c	-0.41	1.12E-08	2.67E-07
DNA	TcMar-Tc2	Kanga1d	-0.27	1.08E-05	5.32E-05
DNA	TcMar-Tc2	Kanga2_a	-0.16	5.55E-04	1.30E-03
DNA	TcMar-Tc2	MER104	-0.13	4.76E-03	8.11E-03
DNA	TcMar-Tigger	MamRep137	-0.29	2.06E-07	2.36E-06
DNA	TcMar-Tigger	MER127	-0.55	3.42E-09	1.12E-07
DNA	TcMar-Tigger	MER2	-0.15	1.67E-03	3.34E-03
DNA	TcMar-Tigger	MER44A	-0.17	4.50E-04	1.09E-03

DNA	TcMar-Tigger	MER44B	-0.21	5.19E-05	1.91E-04
DNA	TcMar-Tigger	MER44C	-0.15	2.46E-03	4.61E-03
DNA	TcMar-Tigger	MER44D	-0.29	7.52E-07	6.51E-06
DNA	TcMar-Tigger	MER47A	-0.18	1.17E-04	3.66E-04
DNA	TcMar-Tigger	MER47B	-0.34	9.57E-09	2.47E-07
DNA	TcMar-Tigger	MER47C	-0.25	7.37E-05	2.49E-04
DNA	TcMar-Tigger	MER6	-0.23	9.57E-06	4.75E-05
DNA	TcMar-Tigger	MER8	-0.14	1.84E-03	3.60E-03
DNA	TcMar-Tigger	MERX	-0.18	2.73E-03	5.02E-03
DNA	TcMar-Tigger	Tigger1	-0.18	3.57E-04	9.07E-04
DNA	TcMar-Tigger	Tigger10	-0.2	5.07E-05	1.87E-04
DNA	TcMar-Tigger	Tigger12c	-0.14	2.88E-03	5.25E-03
DNA	TcMar-Tigger	Tigger13a	-0.19	1.14E-04	3.60E-04
DNA	TcMar-Tigger	Tigger14a	-0.16	1.31E-03	2.71E-03
DNA	TcMar-Tigger	Tigger15a	-0.19	1.90E-04	5.44E-04
DNA	TcMar-Tigger	Tigger16a	-0.22	8.80E-06	4.45E-05
DNA	TcMar-Tigger	Tigger16b	-0.2	3.27E-05	1.31E-04
DNA	TcMar-Tigger	Tigger17	-0.26	1.67E-03	3.34E-03
DNA	TcMar-Tigger	Tigger17a	-0.19	1.54E-04	4.62E-04
DNA	TcMar-Tigger	Tigger17b	-0.47	1.64E-05	7.35E-05
DNA	TcMar-Tigger	Tigger17c	-0.16	1.71E-03	3.39E-03
DNA	TcMar-Tigger	Tigger18a	-0.17	7.33E-04	1.66E-03
DNA	TcMar-Tigger	Tigger1a_Art	-0.21	2.79E-03	5.12E-03
DNA	TcMar-Tigger	Tigger1a_Mars	-0.73	1.39E-06	1.04E-05
DNA	TcMar-Tigger	Tigger2	-0.17	5.05E-04	1.20E-03
DNA	TcMar-Tigger	Tigger20a	-0.2	9.52E-05	3.11E-04
DNA	TcMar-Tigger	Tigger2a	-0.16	1.01E-03	2.15E-03
DNA	TcMar-Tigger	Tigger2b	-0.42	2.23E-04	6.15E-04
DNA	TcMar-Tigger	Tigger3	-0.27	1.72E-06	1.22E-05
DNA	TcMar-Tigger	Tigger3a	-0.14	2.71E-03	5.01E-03
DNA	TcMar-Tigger	Tigger3b	-0.18	5.55E-04	1.30E-03
DNA	TcMar-Tigger	Tigger3c	-0.16	8.88E-04	1.94E-03
DNA	TcMar-Tigger	Tigger3d	-0.19	1.17E-03	2.46E-03
DNA	TcMar-Tigger	Tigger4	-0.2	3.71E-05	1.44E-04
DNA	TcMar-Tigger	Tigger4b	-0.17	2.57E-04	6.95E-04
DNA	TcMar-Tigger	Tigger5	-0.15	3.75E-03	6.54E-03
DNA	TcMar-Tigger	Tigger5b	-0.14	3.64E-03	6.39E-03
DNA	TcMar-Tigger	Tigger6a	-0.16	1.72E-03	3.41E-03
DNA	TcMar-Tigger	Tigger6b	-0.26	4.47E-04	1.09E-03
DNA	TcMar-Tigger	Tigger7	-0.18	1.84E-04	5.32E-04
DNA	TcMar?	MER121	-0.16	4.24E-03	7.33E-03
LINE	CR1	L3	-0.17	9.82E-04	2.10E-03
LINE	CR1	L3b	-0.19	1.37E-04	4.20E-04
LINE	CR1	Plat_L3	-0.25	2.34E-06	1.55E-05
LINE	CR1	X5A_LINE	-0.46	4.76E-04	1.15E-03
LINE	CR1	X6A_LINE	-0.47	7.23E-06	3.85E-05
LINE	CR1	X7B_LINE	-0.2	3.95E-04	9.84E-04
LINE	Dong-R4	Mam_R4	-0.18	2.43E-03	4.57E-03
LINE	L1	HAL1b	-0.14	3.53E-03	6.24E-03
LINE	L1	HAL1ME	-0.22	2.38E-05	9.94E-05

LINE	L1	L1HS	-0.24	7.81E-06	4.05E-05
LINE	L1	L1M2a1	-0.57	1.12E-10	8.29E-09
LINE	L1	L1M3a	-0.2	4.45E-04	1.09E-03
LINE	L1	L1M3e	-0.23	2.19E-05	9.38E-05
LINE	L1	L1M4a1	-0.15	3.06E-03	5.51E-03
LINE	L1	L1M6B	-0.19	1.99E-04	5.60E-04
LINE	L1	L1M7	-0.25	4.94E-06	2.82E-05
LINE	L1	L1M8	-0.26	2.35E-06	1.55E-05
LINE	L1	L1MA1	-0.18	8.64E-04	1.90E-03
LINE	L1	L1MA4	-0.16	2.56E-03	4.76E-03
LINE	L1	L1MB1	-0.18	3.68E-04	9.31E-04
LINE	L1	L1MB8	-0.15	4.89E-03	8.30E-03
LINE	L1	L1MC1	-0.18	6.57E-04	1.51E-03
LINE	L1	L1MC4	-0.17	9.43E-04	2.04E-03
LINE	L1	L1MC4a	-0.16	2.20E-03	4.24E-03
LINE	L1	L1MCb	-0.21	3.98E-05	1.53E-04
LINE	L1	L1MCc	-0.16	7.51E-04	1.69E-03
LINE	L1	L1MD	-0.19	2.45E-04	6.70E-04
LINE	L1	L1MD3	-0.15	2.07E-03	4.02E-03
LINE	L1	L1MDa	-0.15	3.91E-03	6.80E-03
LINE	L1	L1ME3A	-0.18	3.91E-04	9.78E-04
LINE	L1	L1ME3B	-0.18	3.88E-04	9.75E-04
LINE	L1	L1ME3D	-0.16	1.53E-03	3.08E-03
LINE	L1	L1ME3E	-0.16	1.52E-03	3.07E-03
LINE	L1	L1ME4b	-0.16	1.74E-03	3.42E-03
LINE	L1	L1ME4c	-0.14	4.63E-03	7.94E-03
LINE	L1	L1ME5	-0.14	4.22E-03	7.31E-03
LINE	L1	L1MEc	-0.17	1.34E-03	2.77E-03
LINE	L1	L1MEd	-0.18	4.08E-04	1.01E-03
LINE	L1	L1MEg1	-0.15	4.41E-03	7.60E-03
LINE	L1	L1MEi	-0.17	7.82E-04	1.75E-03
LINE	L1	L1MEj	-0.25	7.53E-06	3.96E-05
LINE	L1	L1P1	-0.26	3.24E-06	1.97E-05
LINE	L1	L1P2	-0.23	2.03E-05	8.79E-05
LINE	L1	L1P4b	-0.4	5.47E-05	1.98E-04
LINE	L1	L1P4c	-0.6	2.42E-03	4.56E-03
LINE	L1	L1PA10	-0.16	2.54E-03	4.74E-03
LINE	L1	L1PA12	-0.38	2.51E-09	8.94E-08
LINE	L1	L1PA2	-0.21	1.43E-04	4.34E-04
LINE	L1	L1PA3	-0.26	1.14E-05	5.53E-05
LINE	L1	L1PA4	-0.26	8.95E-06	4.51E-05
LINE	L1	L1PA5	-0.23	5.26E-05	1.92E-04
LINE	L1	L1PA6	-0.15	2.51E-03	4.69E-03
LINE	L1	L1PA7	-0.17	2.31E-03	4.41E-03
LINE	L1	L1PA8	-0.17	1.87E-03	3.67E-03
LINE	L1	L1PBa1	-0.22	4.90E-05	1.84E-04
LINE	L2	L2	-0.18	9.40E-04	2.03E-03
LINE	L2	L2b	-0.15	4.72E-03	8.07E-03
LINE	L2	L2c	-0.18	8.24E-04	1.83E-03
LINE	RTE-BovB	X3_LINE	-0.18	2.21E-03	4.25E-03

LINE	RTE-X	L4_A_Mam	-0.19	1.23E-04	3.82E-04
LINE	RTE-X	L4_B_Mam	-0.17	2.25E-04	6.18E-04
LINE	RTE-X	L4_C_Mam	-0.21	3.28E-05	1.31E-04
LINE	RTE-X	L5	-0.13	4.38E-03	7.57E-03
LTR	ERV1	ERV24B_Prim-int	-0.19	9.66E-04	2.08E-03
LTR	ERV1	Harlequin-int	-0.15	2.08E-03	4.04E-03
LTR	ERV1	HERV-Fc1_LTR1	-1.02	5.14E-06	2.89E-05
LTR	ERV1	HERV-Fc1-int	-1.17	2.06E-14	5.31E-12
LTR	ERV1	HERV1_I-int	-0.22	2.11E-03	4.08E-03
LTR	ERV1	HERV1_LTRa	-0.49	3.23E-03	5.77E-03
LTR	ERV1	HERV1_LTRb	-0.34	1.34E-03	2.77E-03
LTR	ERV1	HERV15-int	-0.49	7.16E-07	6.30E-06
LTR	ERV1	HERV30-int	-0.24	5.98E-03	9.94E-03
LTR	ERV1	HERV4_I-int	-0.18	1.09E-03	2.30E-03
LTR	ERV1	HERVE_a-int	-0.16	3.75E-03	6.54E-03
LTR	ERV1	HERVE-int	-0.48	1.99E-13	3.42E-11
LTR	ERV1	HERVFH21-int	-0.32	7.77E-05	2.59E-04
LTR	ERV1	HERVH-int	-0.33	6.15E-07	5.88E-06
LTR	ERV1	HERVH48-int	-0.27	1.42E-04	4.33E-04
LTR	ERV1	HERVI-int	-0.62	3.06E-07	3.33E-06
LTR	ERV1	HERVP71A-int	-0.39	1.97E-08	3.91E-07
LTR	ERV1	HERVS71-int	-0.61	8.23E-10	4.04E-08
LTR	ERV1	HUERS-P2-int	-0.25	1.41E-03	2.88E-03
LTR	ERV1	HUERS-P3-int	-0.21	5.68E-05	2.03E-04
LTR	ERV1	HUERS-P3b-int	-0.3	8.86E-08	1.24E-06
LTR	ERV1	LTR06	-0.59	7.57E-07	6.51E-06
LTR	ERV1	LTR1	-0.4	3.38E-07	3.54E-06
LTR	ERV1	LTR10A	-0.27	1.43E-06	1.07E-05
LTR	ERV1	LTR10B2	-0.97	6.83E-06	3.71E-05
LTR	ERV1	LTR10C	-0.21	1.08E-04	3.46E-04
LTR	ERV1	LTR10D	-0.24	5.70E-03	9.55E-03
LTR	ERV1	LTR10E	-0.24	3.14E-05	1.27E-04
LTR	ERV1	LTR10F	-0.15	5.33E-03	8.98E-03
LTR	ERV1	LTR10G	-0.24	3.02E-03	5.46E-03
LTR	ERV1	LTR12	-0.19	2.20E-04	6.08E-04
LTR	ERV1	LTR12_	-0.34	1.13E-07	1.42E-06
LTR	ERV1	LTR12B	-0.3	5.62E-05	2.02E-04
LTR	ERV1	LTR12D	-0.15	2.59E-03	4.81E-03
LTR	ERV1	LTR12E	-0.31	2.86E-05	1.18E-04
LTR	ERV1	LTR12F	-0.27	3.08E-06	1.91E-05
LTR	ERV1	LTR15	-0.25	9.30E-04	2.02E-03
LTR	ERV1	LTR19A	-0.33	3.64E-06	2.17E-05
LTR	ERV1	LTR19C	-0.29	1.96E-05	8.54E-05
LTR	ERV1	LTR1A1	-0.26	8.20E-05	2.69E-04
LTR	ERV1	LTR1A2	-0.17	1.34E-03	2.77E-03
LTR	ERV1	LTR1B	-0.21	2.36E-03	4.46E-03
LTR	ERV1	LTR1B0	-0.44	8.64E-05	2.83E-04
LTR	ERV1	LTR1C	-0.42	1.33E-04	4.08E-04
LTR	ERV1	LTR1C1	-0.37	1.35E-03	2.77E-03
LTR	ERV1	LTR1D	-0.3	5.95E-05	2.10E-04

LTR	ERV1	LTR1D1	-0.31	7.30E-06	3.86E-05
LTR	ERV1	LTR1E	-0.4	6.66E-08	9.96E-07
LTR	ERV1	LTR1F1	-0.24	2.83E-03	5.16E-03
LTR	ERV1	LTR1F2	-0.31	3.32E-04	8.55E-04
LTR	ERV1	LTR21A	-0.21	1.44E-03	2.93E-03
LTR	ERV1	LTR24	-0.47	2.69E-06	1.74E-05
LTR	ERV1	LTR24C	-0.34	1.14E-05	5.54E-05
LTR	ERV1	LTR26	-0.23	6.72E-05	2.30E-04
LTR	ERV1	LTR26B	-0.33	9.59E-04	2.07E-03
LTR	ERV1	LTR26E	-0.51	2.91E-07	3.26E-06
LTR	ERV1	LTR27	-0.38	1.13E-04	3.58E-04
LTR	ERV1	LTR2752	-0.24	3.31E-03	5.89E-03
LTR	ERV1	LTR27B	-0.26	2.96E-03	5.38E-03
LTR	ERV1	LTR28	-0.36	7.62E-06	3.97E-05
LTR	ERV1	LTR28B	-0.26	2.53E-04	6.86E-04
LTR	ERV1	LTR28C	-0.33	4.47E-08	7.32E-07
LTR	ERV1	LTR2B	-0.16	3.16E-03	5.67E-03
LTR	ERV1	LTR2C	-0.25	8.03E-05	2.66E-04
LTR	ERV1	LTR30	-0.38	6.71E-05	2.30E-04
LTR	ERV1	LTR31	-0.26	1.60E-04	4.79E-04
LTR	ERV1	LTR35A	-0.44	1.17E-06	9.25E-06
LTR	ERV1	LTR37A	-0.19	1.13E-04	3.58E-04
LTR	ERV1	LTR37B	-0.21	3.40E-05	1.34E-04
LTR	ERV1	LTR38A1	-0.5	5.02E-06	2.83E-05
LTR	ERV1	LTR38B	-0.37	2.31E-05	9.77E-05
LTR	ERV1	LTR38C	-0.2	5.66E-03	9.51E-03
LTR	ERV1	LTR39-int	-0.31	3.53E-04	8.99E-04
LTR	ERV1	LTR43	-0.18	3.57E-03	6.29E-03
LTR	ERV1	LTR45	-0.35	4.99E-05	1.86E-04
LTR	ERV1	LTR45B	-0.18	2.77E-03	5.10E-03
LTR	ERV1	LTR46	-0.5	2.47E-09	8.94E-08
LTR	ERV1	LTR46-int	-0.63	3.87E-11	3.52E-09
LTR	ERV1	LTR48B	-0.33	3.59E-08	6.17E-07
LTR	ERV1	LTR49	-0.22	2.99E-05	1.22E-04
LTR	ERV1	LTR49-int	-0.14	5.90E-03	9.83E-03
LTR	ERV1	LTR56	-0.31	7.22E-08	1.05E-06
LTR	ERV1	LTR60	-0.41	6.86E-04	1.57E-03
LTR	ERV1	LTR60B	-0.32	2.64E-04	7.09E-04
LTR	ERV1	LTR65	-0.25	5.35E-05	1.95E-04
LTR	ERV1	LTR6A	-0.25	1.73E-05	7.66E-05
LTR	ERV1	LTR6B	-0.54	1.05E-08	2.57E-07
LTR	ERV1	LTR7	-0.3	1.55E-07	1.88E-06
LTR	ERV1	LTR70	-0.5	2.30E-11	2.37E-09
LTR	ERV1	LTR71A	-0.27	1.70E-04	5.01E-04
LTR	ERV1	LTR71B	-0.73	6.04E-14	1.25E-11
LTR	ERV1	LTR72	-0.3	4.27E-04	1.05E-03
LTR	ERV1	LTR72B	-0.25	2.42E-03	4.56E-03
LTR	ERV1	LTR73	-0.27	4.40E-04	1.08E-03
LTR	ERV1	LTR76	-0.37	1.79E-08	3.63E-07
LTR	ERV1	LTR77	-0.87	2.16E-11	2.37E-09

LTR	ERV1	LTR78	-0.23	1.12E-05	5.47E-05
LTR	ERV1	LTR78B	-0.31	1.50E-08	3.30E-07
LTR	ERV1	LTR7B	-0.36	1.76E-08	3.63E-07
LTR	ERV1	LTR7C	-0.3	1.60E-06	1.17E-05
LTR	ERV1	LTR7Y	-0.37	4.12E-08	6.97E-07
LTR	ERV1	LTR8	-0.17	5.36E-04	1.26E-03
LTR	ERV1	LTR8A	-0.31	9.07E-08	1.24E-06
LTR	ERV1	LTR9	-0.24	4.82E-06	2.78E-05
LTR	ERV1	LTR9A1	-0.48	6.53E-10	3.49E-08
LTR	ERV1	LTR9B	-0.2	3.18E-04	8.22E-04
LTR	ERV1	LTR9C	-0.26	8.57E-04	1.89E-03
LTR	ERV1	LTR9D	-0.31	1.11E-07	1.42E-06
LTR	ERV1	MER101-int	-0.14	4.83E-03	8.21E-03
LTR	ERV1	MER101B	-0.24	8.56E-04	1.89E-03
LTR	ERV1	MER110	-0.37	6.73E-07	6.13E-06
LTR	ERV1	MER110-int	-0.31	1.77E-05	7.76E-05
LTR	ERV1	MER31-int	-0.29	1.58E-07	1.88E-06
LTR	ERV1	MER31A	-0.25	3.77E-06	2.24E-05
LTR	ERV1	MER31B	-0.21	1.62E-04	4.82E-04
LTR	ERV1	MER34	-0.13	5.78E-03	9.64E-03
LTR	ERV1	MER34B	-0.23	2.00E-04	5.60E-04
LTR	ERV1	MER34B-int	0.29	4.52E-07	4.57E-06
LTR	ERV1	MER34C2	-0.22	6.79E-04	1.56E-03
LTR	ERV1	MER39	-0.18	4.90E-04	1.17E-03
LTR	ERV1	MER39B	-0.37	6.77E-10	3.49E-08
LTR	ERV1	MER41A	-0.22	3.01E-05	1.22E-04
LTR	ERV1	MER41B	-0.19	9.65E-05	3.13E-04
LTR	ERV1	MER41C	-0.14	5.68E-03	9.53E-03
LTR	ERV1	MER41D	-0.27	9.17E-06	4.59E-05
LTR	ERV1	MER41E	-0.25	1.99E-04	5.60E-04
LTR	ERV1	MER49	-0.25	1.26E-06	9.62E-06
LTR	ERV1	MER4A1	-0.22	5.39E-05	1.96E-04
LTR	ERV1	MER4A1_	-0.17	1.27E-03	2.64E-03
LTR	ERV1	MER4D	-0.17	2.71E-04	7.21E-04
LTR	ERV1	MER4D0	-0.19	1.45E-03	2.95E-03
LTR	ERV1	MER4D1	-0.19	3.07E-04	7.98E-04
LTR	ERV1	MER4E	-0.29	9.49E-07	7.78E-06
LTR	ERV1	MER4E1	-0.24	1.63E-05	7.35E-05
LTR	ERV1	MER50B	-0.25	2.14E-05	9.22E-05
LTR	ERV1	MER50C	-0.33	7.15E-04	1.63E-03
LTR	ERV1	MER51C	0.64	1.65E-05	7.38E-05
LTR	ERV1	MER52A	-0.32	2.24E-08	4.35E-07
LTR	ERV1	MER52C	-0.22	1.14E-03	2.40E-03
LTR	ERV1	MER52D	-0.21	2.29E-03	4.37E-03
LTR	ERV1	MER57-int	-0.18	7.25E-04	1.65E-03
LTR	ERV1	MER57A-int	-0.2	6.45E-05	2.23E-04
LTR	ERV1	MER57A1	-0.15	2.33E-03	4.43E-03
LTR	ERV1	MER57B1	-0.17	9.35E-04	2.03E-03
LTR	ERV1	MER57B2	-0.25	8.25E-06	4.21E-05
LTR	ERV1	MER57D	-0.18	2.44E-03	4.58E-03

LTR	ERV1	MER57E2	-0.7	6.90E-07	6.19E-06
LTR	ERV1	MER57E3	-0.36	3.39E-07	3.54E-06
LTR	ERV1	MER57F	-0.27	1.74E-04	5.12E-04
LTR	ERV1	MER61A	-0.32	8.09E-07	6.82E-06
LTR	ERV1	MER61C	-0.39	1.93E-06	1.33E-05
LTR	ERV1	MER61D	-0.32	5.41E-06	2.99E-05
LTR	ERV1	MER61E	-0.5	1.24E-07	1.54E-06
LTR	ERV1	MER65B	0.61	1.06E-07	1.38E-06
LTR	ERV1	MER65D	0.19	2.18E-03	4.21E-03
LTR	ERV1	MER66B	-0.21	5.54E-05	1.99E-04
LTR	ERV1	MER66C	-0.29	9.76E-06	4.82E-05
LTR	ERV1	MER66D	-0.28	9.61E-05	3.13E-04
LTR	ERV1	MER67B	-0.3	1.27E-06	9.66E-06
LTR	ERV1	MER67D	-0.48	1.21E-09	5.53E-08
LTR	ERV1	MER72	-0.31	8.68E-07	7.22E-06
LTR	ERV1	MER83	-0.21	2.05E-04	5.72E-04
LTR	ERV1	MER83B	-0.42	2.04E-09	8.42E-08
LTR	ERV1	MER83B-int	-0.3	1.48E-05	6.94E-05
LTR	ERV1	MER84	0.21	1.05E-04	3.39E-04
LTR	ERV1	MER84-int	-0.55	4.42E-09	1.23E-07
LTR	ERV1	MER87	-0.43	3.13E-07	3.33E-06
LTR	ERV1	MER87B	-0.23	7.39E-04	1.67E-03
LTR	ERV1	MER89	-0.2	5.71E-05	2.03E-04
LTR	ERV1	MER89-int	-0.32	1.70E-06	1.22E-05
LTR	ERV1	MER90a	-0.17	4.81E-04	1.15E-03
LTR	ERV1	MER92B	-0.17	7.27E-04	1.65E-03
LTR	ERV1	MER95	-0.53	2.67E-06	1.73E-05
LTR	ERV1	PABL_A	-0.22	2.58E-04	6.95E-04
LTR	ERV1	PABL_B	-0.19	2.64E-03	4.89E-03
LTR	ERV1	PABL_B-int	-0.43	3.24E-09	1.12E-07
LTR	ERV1	PRIMA4_LTR	-0.35	7.13E-06	3.81E-05
LTR	ERV1	PRIMA4-int	-0.38	3.90E-09	1.18E-07
LTR	ERV1	PRIMA41-int	-0.3	8.74E-07	7.22E-06
LTR	ERV1	PRIMAX-int	-0.42	1.00E-03	2.13E-03
LTR	ERV1	PrimLTR79	-0.26	1.70E-03	3.38E-03
LTR	ERV1?	LTR103_Mam	-0.34	1.59E-05	7.24E-05
LTR	ERV1?	LTR103b_Mam	-0.17	5.10E-03	8.60E-03
LTR	ERVK	HERVK-int	-0.68	1.42E-15	4.93E-13
LTR	ERVK	HERVK11-int	-0.92	4.62E-11	3.67E-09
LTR	ERVK	HERVK11D-int	0.38	1.94E-04	5.51E-04
LTR	ERVK	HERVK13-int	-0.46	4.16E-10	2.53E-08
LTR	ERVK	HERVK14C-int	0.38	7.74E-04	1.73E-03
LTR	ERVK	HERVK22-int	-0.26	1.63E-06	1.18E-05
LTR	ERVK	HERVK3-int	-0.44	3.93E-12	5.80E-10
LTR	ERVK	HERVK9-int	-0.34	3.46E-09	1.12E-07
LTR	ERVK	LTR13	-0.16	1.67E-03	3.34E-03
LTR	ERVK	LTR13_	-0.2	5.97E-03	9.94E-03
LTR	ERVK	LTR14B	-0.37	6.98E-07	6.21E-06
LTR	ERVK	LTR22	-0.5	6.70E-05	2.30E-04
LTR	ERVK	LTR22B	-0.32	1.56E-05	7.18E-05

LTR	ERVK	LTR22B1	-0.4	1.64E-05	7.35E-05
LTR	ERVK	LTR22B2	-0.95	4.29E-08	7.15E-07
LTR	ERVK	LTR22C	-0.79	6.21E-05	2.18E-04
LTR	ERVK	LTR22C0	-0.46	8.12E-05	2.68E-04
LTR	ERVK	LTR22C2	-0.67	4.32E-09	1.23E-07
LTR	ERVK	LTR22E	-0.21	5.21E-04	1.24E-03
LTR	ERVK	LTR3	-0.31	2.29E-05	9.73E-05
LTR	ERVK	LTR3A	-0.35	4.79E-07	4.76E-06
LTR	ERVK	LTR3B	-0.34	1.70E-04	5.01E-04
LTR	ERVK	LTR3B_	-0.28	1.49E-04	4.50E-04
LTR	ERVK	LTR5_Hs	-0.22	1.18E-05	5.60E-05
LTR	ERVK	LTR5A	-0.62	1.13E-15	4.93E-13
LTR	ERVK	LTR5B	-0.26	5.88E-07	5.73E-06
LTR	ERVK	MER11A	-0.24	8.56E-06	4.35E-05
LTR	ERVK	MER11B	-0.19	1.20E-04	3.75E-04
LTR	ERVK	MER11C	-0.31	5.91E-08	9.10E-07
LTR	ERVK	MER9a1	-0.23	4.30E-04	1.06E-03
LTR	ERVK	MER9a2	-0.21	1.59E-04	4.75E-04
LTR	ERVK	MER9B	-0.49	5.17E-04	1.23E-03
LTR	ERVL	ERV3-16A3_I-int	-0.3	5.05E-08	7.99E-07
LTR	ERVL	ERV3-16A3_LTR	-0.33	8.13E-07	6.82E-06
LTR	ERVL	ERVL-B4-int	-0.16	1.04E-03	2.20E-03
LTR	ERVL	ERVL-E-int	-0.26	1.31E-06	9.86E-06
LTR	ERVL	ERVL-int	-0.35	1.14E-08	2.67E-07
LTR	ERVL	ERVL47-int	-0.42	3.85E-05	1.48E-04
LTR	ERVL	HERVL18-int	-0.26	5.50E-05	1.98E-04
LTR	ERVL	HERVL32-int	-0.97	1.43E-15	4.93E-13
LTR	ERVL	HERVL40-int	-0.33	5.01E-09	1.36E-07
LTR	ERVL	HERVL66-int	-0.3	2.47E-04	6.73E-04
LTR	ERVL	LTR101_Mam	-0.17	2.57E-03	4.79E-03
LTR	ERVL	LTR102_Mam	-0.24	3.25E-04	8.38E-04
LTR	ERVL	LTR105_Mam	-0.41	9.15E-08	1.24E-06
LTR	ERVL	LTR108a_Mam	-0.37	3.38E-04	8.69E-04
LTR	ERVL	LTR16	-0.21	1.80E-04	5.28E-04
LTR	ERVL	LTR16A	-0.29	9.32E-08	1.25E-06
LTR	ERVL	LTR16A1	-0.15	3.62E-03	6.36E-03
LTR	ERVL	LTR16A2	-0.17	1.69E-03	3.36E-03
LTR	ERVL	LTR16B	-0.38	2.98E-07	3.30E-06
LTR	ERVL	LTR16B1	-0.35	2.38E-08	4.55E-07
LTR	ERVL	LTR16B2	-0.22	7.29E-05	2.47E-04
LTR	ERVL	LTR16C	-0.25	5.60E-06	3.07E-05
LTR	ERVL	LTR16D1	-0.3	8.27E-04	1.83E-03
LTR	ERVL	LTR16D2	-0.49	1.54E-08	3.31E-07
LTR	ERVL	LTR16E1	-0.28	6.67E-07	6.13E-06
LTR	ERVL	LTR18A	-0.49	1.23E-09	5.53E-08
LTR	ERVL	LTR18B	-0.24	2.07E-04	5.75E-04
LTR	ERVL	LTR18C	-0.5	7.17E-04	1.63E-03
LTR	ERVL	LTR32	-0.25	7.92E-06	4.08E-05
LTR	ERVL	LTR33	-0.28	3.60E-07	3.67E-06
LTR	ERVL	LTR33A	-0.18	1.83E-04	5.32E-04

LTR	ERVL	LTR33A_	-0.31	1.41E-07	1.74E-06
LTR	ERVL	LTR33B	-0.27	6.07E-07	5.86E-06
LTR	ERVL	LTR33C	-0.18	3.83E-04	9.66E-04
LTR	ERVL	LTR40a	-0.23	2.21E-05	9.42E-05
LTR	ERVL	LTR40A1	-0.26	5.69E-04	1.32E-03
LTR	ERVL	LTR40b	-0.28	4.90E-07	4.81E-06
LTR	ERVL	LTR40c	-0.18	5.78E-04	1.34E-03
LTR	ERVL	LTR41	-0.22	2.35E-05	9.86E-05
LTR	ERVL	LTR41B	-0.17	9.17E-04	2.00E-03
LTR	ERVL	LTR41C	-0.31	6.82E-08	1.01E-06
LTR	ERVL	LTR42	-0.23	2.67E-04	7.14E-04
LTR	ERVL	LTR47A	-0.24	3.11E-05	1.26E-04
LTR	ERVL	LTR47A2	-0.25	3.91E-04	9.78E-04
LTR	ERVL	LTR47B2	-0.71	1.04E-08	2.57E-07
LTR	ERVL	LTR47B3	-0.36	6.32E-04	1.46E-03
LTR	ERVL	LTR52	-0.28	1.68E-06	1.21E-05
LTR	ERVL	LTR53	-0.5	2.88E-10	1.86E-08
LTR	ERVL	LTR53B	-0.44	1.17E-05	5.60E-05
LTR	ERVL	LTR57	0.36	3.12E-07	3.33E-06
LTR	ERVL	LTR57-int	-0.34	2.93E-06	1.84E-05
LTR	ERVL	LTR66	-0.32	1.57E-05	7.20E-05
LTR	ERVL	LTR69	-0.28	1.89E-03	3.70E-03
LTR	ERVL	LTR75	-0.25	1.30E-04	4.03E-04
LTR	ERVL	LTR75B	-0.47	6.86E-06	3.71E-05
LTR	ERVL	LTR79	-0.25	2.35E-06	1.55E-05
LTR	ERVL	LTR80B	-0.43	1.20E-08	2.75E-07
LTR	ERVL	LTR82B	-0.19	4.54E-04	1.10E-03
LTR	ERVL	LTR83	-0.51	4.09E-11	3.52E-09
LTR	ERVL	LTR84b	-0.29	1.58E-07	1.88E-06
LTR	ERVL	LTR86A1	-0.16	2.03E-03	3.96E-03
LTR	ERVL	LTR86A2	-0.36	1.12E-07	1.42E-06
LTR	ERVL	LTR86B2	-0.21	4.90E-03	8.30E-03
LTR	ERVL	LTR86C	-0.2	4.98E-03	8.43E-03
LTR	ERVL	LTR91	-0.5	1.90E-06	1.33E-05
LTR	ERVL	MER21-int	-0.18	3.58E-04	9.07E-04
LTR	ERVL	MER21C	-0.15	5.71E-03	9.56E-03
LTR	ERVL	MER54A	-0.28	7.21E-07	6.30E-06
LTR	ERVL	MER68	-0.24	3.25E-06	1.97E-05
LTR	ERVL	MER68-int	-0.29	2.87E-06	1.82E-05
LTR	ERVL	MER68B	-0.29	1.62E-07	1.90E-06
LTR	ERVL	MER68C	-0.22	3.78E-03	6.60E-03
LTR	ERVL	MER70A	-0.29	1.50E-05	6.98E-05
LTR	ERVL	MER73	-0.27	4.78E-05	1.81E-04
LTR	ERVL	MER74A	-0.34	3.57E-08	6.17E-07
LTR	ERVL	MER74B	-0.2	2.73E-04	7.24E-04
LTR	ERVL	MER74C	-0.61	3.12E-08	5.55E-07
LTR	ERVL	MER76-int	-0.56	2.49E-09	8.94E-08
LTR	ERVL	MER77	-0.15	2.23E-03	4.27E-03
LTR	ERVL	MER77B	-0.38	1.33E-09	5.72E-08
LTR	ERVL	MLT2A1	-0.31	9.69E-07	7.88E-06

LTR	ERVL	MLT2A2	-0.33	2.46E-08	4.62E-07
LTR	ERVL	MLT2B1	-0.17	1.25E-03	2.62E-03
LTR	ERVL	MLT2B2	-0.25	5.30E-06	2.94E-05
LTR	ERVL	MLT2B3	-0.27	1.52E-06	1.12E-05
LTR	ERVL	MLT2B4	-0.22	3.56E-05	1.40E-04
LTR	ERVL	MLT2B5	-0.3	5.04E-05	1.87E-04
LTR	ERVL	MLT2C1	-0.15	3.28E-03	5.85E-03
LTR	ERVL	MLT2C2	-0.18	5.56E-04	1.30E-03
LTR	ERVL	MLT2D	-0.17	7.94E-04	1.77E-03
LTR	ERVL	MLT2F	-0.29	9.87E-08	1.31E-06
LTR	ERVL-MaLR	MLT1A	-0.2	1.99E-04	5.60E-04
LTR	ERVL-MaLR	MLT1A-int	-0.41	2.04E-07	2.36E-06
LTR	ERVL-MaLR	MLT1A0	-0.22	4.90E-05	1.84E-04
LTR	ERVL-MaLR	MLT1A0-int	-0.4	1.84E-10	1.26E-08
LTR	ERVL-MaLR	MLT1A1	-0.27	1.25E-06	9.62E-06
LTR	ERVL-MaLR	MLT1C-int	-0.23	1.05E-04	3.39E-04
LTR	ERVL-MaLR	MLT1D	-0.17	7.52E-04	1.69E-03
LTR	ERVL-MaLR	MLT1D-int	-0.27	1.06E-06	8.38E-06
LTR	ERVL-MaLR	MLT1E	-0.2	1.87E-04	5.40E-04
LTR	ERVL-MaLR	MLT1E1	-0.19	1.88E-04	5.40E-04
LTR	ERVL-MaLR	MLT1E1A	-0.19	1.91E-04	5.45E-04
LTR	ERVL-MaLR	MLT1E2	-0.24	1.61E-05	7.31E-05
LTR	ERVL-MaLR	MLT1E2-int	-0.33	7.57E-05	2.53E-04
LTR	ERVL-MaLR	MLT1E3	-0.2	7.49E-05	2.52E-04
LTR	ERVL-MaLR	MLT1E3-int	-0.38	2.75E-04	7.28E-04
LTR	ERVL-MaLR	MLT1F	-0.16	1.14E-03	2.39E-03
LTR	ERVL-MaLR	MLT1F-int	-0.16	2.95E-03	5.37E-03
LTR	ERVL-MaLR	MLT1F1	-0.2	6.99E-05	2.38E-04
LTR	ERVL-MaLR	MLT1F1-int	-0.17	2.15E-03	4.15E-03
LTR	ERVL-MaLR	MLT1F2	-0.19	1.97E-04	5.58E-04
LTR	ERVL-MaLR	MLT1F2-int	-0.31	2.34E-05	9.84E-05
LTR	ERVL-MaLR	MLT1G	-0.25	2.97E-06	1.86E-05
LTR	ERVL-MaLR	MLT1G-int	-0.37	1.36E-05	6.39E-05
LTR	ERVL-MaLR	MLT1G1	-0.21	3.67E-05	1.43E-04
LTR	ERVL-MaLR	MLT1G1-int	-0.26	4.51E-04	1.09E-03
LTR	ERVL-MaLR	MLT1G3	-0.15	1.77E-03	3.48E-03
LTR	ERVL-MaLR	MLT1H	-0.22	1.18E-05	5.60E-05
LTR	ERVL-MaLR	MLT1H1-int	-0.52	2.21E-09	8.78E-08
LTR	ERVL-MaLR	MLT1H2	-0.17	4.37E-04	1.07E-03
LTR	ERVL-MaLR	MLT1H2-int	-0.44	7.72E-08	1.11E-06
LTR	ERVL-MaLR	MLT1I	-0.21	2.93E-05	1.20E-04
LTR	ERVL-MaLR	MLT1J	-0.16	1.46E-03	2.96E-03
LTR	ERVL-MaLR	MLT1J1	-0.17	5.37E-04	1.26E-03
LTR	ERVL-MaLR	MLT1J1-int	-0.25	6.23E-05	2.18E-04
LTR	ERVL-MaLR	MLT1J2	-0.27	2.21E-07	2.50E-06
LTR	ERVL-MaLR	MLT1J2-int	-0.31	5.26E-06	2.94E-05
LTR	ERVL-MaLR	MLT1K	-0.23	1.16E-05	5.58E-05
LTR	ERVL-MaLR	MLT1L	-0.18	2.57E-04	6.95E-04
LTR	ERVL-MaLR	MLT1M	-0.19	1.47E-04	4.44E-04
LTR	ERVL-MaLR	MLT1O	-0.22	1.52E-05	7.08E-05

LTR	ERVL-MaLR	MSTA	-0.23	3.58E-05	1.40E-04
LTR	ERVL-MaLR	MSTA1	-0.34	1.79E-08	3.63E-07
LTR	ERVL-MaLR	MSTA1-int	-0.8	1.09E-04	3.46E-04
LTR	ERVL-MaLR	MSTB	-0.24	1.55E-05	7.18E-05
LTR	ERVL-MaLR	MSTB-int	-0.22	4.87E-05	1.84E-04
LTR	ERVL-MaLR	MSTB1	-0.2	1.33E-04	4.08E-04
LTR	ERVL-MaLR	MSTB1-int	-0.25	2.89E-04	7.58E-04
LTR	ERVL-MaLR	MSTB2	-0.15	1.94E-03	3.79E-03
LTR	ERVL-MaLR	MSTB2-int	-0.34	1.42E-04	4.33E-04
LTR	ERVL-MaLR	MSTC	-0.23	1.18E-05	5.60E-05
LTR	ERVL-MaLR	MSTC-int	-0.37	1.84E-06	1.30E-05
LTR	ERVL-MaLR	MSTD	-0.22	3.74E-05	1.44E-04
LTR	ERVL-MaLR	MSTD-int	-0.18	2.76E-04	7.29E-04
LTR	ERVL-MaLR	THE1-int	-0.18	1.05E-03	2.22E-03
LTR	ERVL-MaLR	THE1A	-0.16	1.73E-03	3.41E-03
LTR	ERVL-MaLR	THE1A-int	-0.27	6.41E-07	6.02E-06
LTR	ERVL-MaLR	THE1B	-0.28	6.20E-06	3.38E-05
LTR	ERVL-MaLR	THE1B-int	-0.29	3.56E-07	3.67E-06
LTR	ERVL-MaLR	THE1C	-0.19	4.77E-04	1.15E-03
LTR	ERVL-MaLR	THE1C-int	-0.19	2.81E-03	5.15E-03
LTR	ERVL-MaLR	THE1D	-0.24	2.07E-05	8.92E-05
LTR	ERVL-MaLR	THE1D-int	-0.24	6.99E-06	3.76E-05
LTR	ERVL?	LTR55	-0.29	3.50E-05	1.38E-04
LTR	ERVL?	LTR87	-0.31	5.11E-08	7.99E-07
LTR	Gypsy	LTR104_Mam	-0.33	4.93E-08	7.95E-07
LTR	Gypsy	LTR81A	-0.51	1.69E-11	2.18E-09
LTR	Gypsy	LTR81AB	-0.53	1.74E-05	7.66E-05
LTR	Gypsy	LTR81B	-0.38	3.91E-09	1.18E-07
LTR	Gypsy	LTR81C	-0.35	6.24E-07	5.90E-06
LTR	Gypsy	MamGypLTR1a	-0.48	5.57E-10	3.20E-08
LTR	Gypsy	MamGypLTR1b	-0.3	1.97E-06	1.34E-05
LTR	Gypsy	MamGypLTR1c	-0.28	3.42E-06	2.05E-05
LTR	Gypsy	MamGypLTR1d	-0.3	7.59E-05	2.53E-04
LTR	Gypsy	MamGypLTR2b	-0.4	2.84E-08	5.14E-07
LTR	Gypsy	MamGypLTR3	-0.22	1.66E-04	4.91E-04
LTR	Gypsy?	LTR85b	-0.29	1.24E-06	9.62E-06
LTR	Gypsy?	LTR85c	-0.2	1.91E-04	5.45E-04
LTR	Gypsy?	LTR88b	-0.17	4.11E-03	7.13E-03
LTR	Gypsy?	LTR88c	-0.37	4.92E-06	2.82E-05
LTR	LTR	LTR106_Mam	-0.22	8.37E-04	1.85E-03
LTR	LTR	LTR107_Mam	-0.51	8.74E-08	1.23E-06
LTR	LTR	LTR90B	-0.29	6.35E-05	2.21E-04
Other	Other	SVA_B	-0.19	5.41E-04	1.27E-03
Other	Other	SVA_C	-0.18	5.86E-04	1.36E-03
Other	Other	SVA_D	-0.21	1.55E-04	4.64E-04
Other	Other	SVA_E	-0.2	2.06E-04	5.72E-04
Other	Other	SVA_F	-0.2	3.51E-04	8.97E-04
RC	Helitron	Helitron1Na_Mam	-0.23	1.54E-03	3.09E-03
RC	Helitron	Helitron3Na_Mam	-0.28	9.79E-07	7.89E-06
RNA	RNA	7SK	-0.54	3.72E-03	6.52E-03

Satellite	acro	ACRO1	-0.61	1.73E-05	7.66E-05
Satellite	centr	ALR_Alpha	-0.35	6.77E-07	6.13E-06
Satellite	centr	SST1	-0.49	2.56E-08	4.72E-07
Satellite	Satellite	_CATTC_n	-0.86	1.09E-04	3.46E-04
Satellite	Satellite	_GAATG_n	-0.74	4.71E-05	1.79E-04
Satellite	Satellite	BSR_Beta	-0.47	4.27E-04	1.05E-03
Satellite	Satellite	CER	-0.34	2.86E-06	1.82E-05
Satellite	Satellite	HSAT5	-0.36	7.83E-05	2.60E-04
Satellite	Satellite	MSR1	0.31	1.36E-03	2.78E-03
Satellite	Satellite	SATR1	-0.37	2.16E-06	1.46E-05
Satellite	Satellite	SATR2	-0.29	2.28E-03	4.37E-03
scRNA	scRNA	HY1	-0.18	2.41E-03	4.56E-03
scRNA	scRNA	HY4	-0.34	2.81E-03	5.15E-03
SINE	Alu	AluYa8	-0.2	4.46E-03	7.65E-03
SINE	Alu	AluYk11	-0.18	1.53E-03	3.08E-03
SINE	Alu	AluYk12	-0.34	4.12E-06	2.41E-05
SINE	Deu	AmnSINE1	-0.15	1.35E-03	2.77E-03
SINE	Deu	AmnSINE2	-0.22	1.83E-04	5.32E-04
SINE	MIR	MIR	-0.15	3.13E-03	5.62E-03
SINE	MIR	MIR3	-0.15	2.29E-03	4.38E-03
SINE	MIR	MIRb	-0.16	3.02E-03	5.46E-03
SINE	MIR	MIRc	-0.15	2.60E-03	4.82E-03
snRNA	snRNA	U13	-0.32	2.59E-04	6.95E-04
snRNA	snRNA	U17	0.93	2.37E-06	1.55E-05
srpRNA	srpRNA	7SLRNA	-0.28	3.27E-05	1.31E-04

Table S9: Results of the family-level pairwise comparison with edgeR. List of all families differentially expressed at FDR ≤ 0.01.

Class	Family	log_2FC	p-value	FDR
DNA	DNA	-0.32	1.82E-08	2.73E-07
DNA	hAT	-0.14	1.92E-03	3.08E-03
DNA	hAT-Blackjack	-0.2	9.25E-07	6.45E-06
DNA	hAT-Charlie	-0.13	2.26E-03	3.51E-03
DNA	hAT-Tip100	-0.13	1.52E-03	2.54E-03
DNA	hAT-Tip100?	-0.18	5.52E-04	1.03E-03
DNA	hAT?	-0.29	1.63E-05	6.10E-05
DNA	Merlin	-0.25	3.06E-03	4.05E-03
DNA	MULE-MuDR	-0.25	1.00E-06	6.45E-06
DNA	PiggyBac	-0.19	9.45E-05	2.24E-04
DNA	PiggyBac?	-0.16	3.77E-02	4.35E-02
DNA	TcMar	0.05	4.80E-01	4.80E-01
DNA	TcMar-Mariner	-0.17	2.14E-05	7.08E-05
DNA	TcMar-Tc2	-0.19	5.75E-06	2.35E-05
DNA	TcMar-Tigger	-0.17	5.87E-05	1.47E-04
DNA	TcMar?	-0.17	2.90E-03	3.96E-03
LINE	CR1	-0.17	2.66E-05	7.98E-05
LINE	Dong-R4	-0.18	2.44E-03	3.55E-03
LINE	L1	-0.12	2.06E-02	2.44E-02
LINE	L1?	-0.17	1.71E-01	1.83E-01
LINE	L2	-0.16	3.90E-04	7.98E-04
LINE	RTE-BovB	-0.18	2.59E-03	3.65E-03
LINE	RTE-X	-0.19	2.96E-06	1.37E-05
LTR	ERV1	-0.12	8.56E-03	1.04E-02
LTR	ERV1?	-0.23	5.36E-05	1.42E-04
LTR	ERVK	-0.29	1.93E-11	8.70E-10
LTR	ERVL	-0.17	1.45E-04	3.11E-04
LTR	ERVL-MaLR	-0.19	2.20E-05	7.08E-05
LTR	ERVL?	-0.13	4.46E-03	5.57E-03
LTR	Gypsy	-0.24	3.73E-08	4.19E-07
LTR	Gypsy?	-0.18	1.09E-04	2.45E-04
LTR	LTR	-0.17	6.71E-04	1.21E-03
Other	Other	-0.18	3.78E-05	1.06E-04
RC	Helitron	-0.15	2.38E-03	3.55E-03
RNA	RNA	-0.54	3.46E-03	4.44E-03
Satellite	acro	-0.61	1.16E-06	6.55E-06
